# Utilisation of dental services by Brazilian adults in rural and urban areas: a multi-group structural equation analysis using the Andersen behavioural model

**DOI:** 10.1186/s12889-020-09100-x

**Published:** 2020-06-17

**Authors:** Fernando José Herkrath, Mario Vianna Vettore, Guilherme Loureiro Werneck

**Affiliations:** 1 Escola Superior de Ciências da Saúde, Universidade do Estado do Amazonas, Av. Carvalho Leal, 1777, Cachoeirinha, Manaus, Amazonas 69065-001 Brazil; 2 Instituto Leônidas e Maria Deane, Fundação Oswaldo Cruz, Rua Teresina, 476, Adrianópolis, Manaus, Amazonas 69057-070 Brazil; 3 Instituto de Medicina Social, Universidade do Estado do Rio de Janeiro, Rua São Francisco Xavier, 524, Maracanã, Bloco D, 7° andar – UERJ, Rio de Janeiro, Rio de Janeiro 20550-013 Brazil; 4 Departamento de Odontologia Social e Preventiva, Faculdade de Odontologia, Universidade Federal de Minas Gerais, Av. Presidente Antônio Carlos, 6627, Pampulha, Belo Horizonte, Minas Gerais 31270-901 Brazil; 5 School of Clinical Dentistry, University of Sheffield, 19 Claremont Crescent, Sheffield, S10 2TA UK; 6 Instituto de Estudos em Saúde Coletiva, Universidade Federal do Rio de Janeiro, Avenida Horácio Macedo, S/N, Ilha do Fundão, Cidade Universitária, Rio de Janeiro, Rio de Janeiro 21941-598 Brazil

**Keywords:** Dental health services utilisation, Health surveys, Theoretical models, Rural population

## Abstract

**Background:**

The utilisation of health services is determined by complex interactions. In this context, rural populations face greater barriers in accessing dental services than do urban populations, and they generally have poorer oral health status. The evaluation of the determinants of health services utilisation is important to support planning and management of dental services. The aim of this study was to evaluate the predictors of dental services utilisation of Brazilian adults living in rural and urban areas.

**Methods:**

Data from 60,202 adults aged 18 years or older who took part in the Brazilian National Health Survey carried out in 2013 were analysed. Predisposing (age, sex, education, social networks), enabling financing (income, durable goods and household’s crowding), enabling organisation (health insurance, registration in primary health care [PHC]) and need variables (eating difficulties, self-perceived tooth loss and self-perceived oral health) were selected based upon the Andersen behavioural model. Multi-group structural equation modeling assessed the direct and indirect associations of independent variables with non-utilisation of dental services and the interval since the last dental visit for individuals living in rural and urban areas.

**Results:**

Adults living in urban areas were more likely to use dental services than those living in rural areas. Lower enabling financing, lower perceived dental needs and lack of PHC registration were directly associated with lower utilisation of dental services (non-utilisation, β = − 0.36, β = − 0.16, β = − 0.03, respectively; and interval since last dental visit, β = 1.25, β = 0.82, β = − 0.12, respectively). The enabling financing (non-utilisation, β_rural_ = − 0.02 [95%CI: − 0.03 to − 0.02], β_urban_ = 0.00 [95%CI: − 0.01 to 0.00]) and PHC registration (non-utilisation, β_rural_ = − 0.03 [95%CI: − 0.04 to − 0.02], β_urban_ = − 0.01 [95%CI, − 0.01 to − 0.01]) non-standardised total effects were stronger in rural areas. Enabling organisation (β = 0.16) and social network (β = − 2.59) latent variables showed a direct effect on the interval since last dental visit in urban areas. Education and social networks influenced utilisation of dental services through different pathways. Males showed less use of dental services in both urban and rural areas (non-utilisation, β_rural_ = − 0.07, β_urban_ = − 0.04; interval since last dental visit, β_rural_ = − 0.07, β_urban_ = − 0.07) and older adults have used dental services longer than younger ones, mainly in rural areas (β_rural_ = 0.26, β_urban_ = 0.17).

**Conclusion:**

Dental services utilisation was lower in rural areas in Brazil. The theoretical model was supported by empirical data and showed different relationships between the predictors in the two geographical contexts. In rural areas, financial aspects, education, primary care availability, sex and age were relevant factors for the utilisation of services.

## Background

The utilisation of health services results from the interaction between contextual and individual factors, including individual characteristics, access to health services and organisation of the health care system [1]. It has been shown that persons living in socially deprived communities, such as rural areas, have less access to health services and poorer health status than those from better-off communities [2, 3]. Rural populations may also experience poorer oral health and limited access to dental care [4–7]. The rate of dental attendance by people living in rural areas is lower and they are more likely to postpone dental appointments due to financial restrictions [8]. Furthermore, seeking care only in an emergency is more common in rural communities [9].

The Brazilian national health care system offers free primary, secondary and tertiary health care, including oral health care, for all age groups. Primary care through family health teams is the preferred gateway to the health system as the usual source of care and this level of care is also responsible for coordinating the healthcare network [10]. Private dental care is also offered to individuals who can purchase private health insurance and to those who can pay dental services directly. The utilization of dental care has increased over recent decades as a result of the expansion of these services. Nevertheless, socially deprived and geographically isolated people still face difficulties in accessing services when compared to those from better-off socioeconomic groups who have fewer health needs [9–14]. This scenario reinforces the inverse equity hypothesis in healthcare utilisation in Brazil where health services are more available and utilised among those who need it less [15].

The relationships between demographic characteristics, socioeconomic factors, health needs, healthcare system organisation, and utilisation of dental services have been investigated [9, 12, 14, 16–19]. However, few studies in Brazil have adopted a theoretical framework to investigate the association of multiple determinants to health services utilisation using robust statistical methods and large samples [17–19]. Studies using samples with national representativeness, theoretical models and adequate analytical approaches that consider the intrinsic and complex interrelationships between the predictors of dental services utilisation are necessary to obtain evidence and new insight on how to tackle the barriers to access dental services [20–23].

Investigating the determinants of dental health services utilisation using data from a nationwide survey can contribute to the identification of opportunities for intervention to reduce dental care inequalities. This study aimed to identify the predisposing, enabling and need-related predictors of dental services utilisation in Brazilian adults using the Andersen behavioural model [24]. The direct and indirect effects between variables were also examined. A specific objective of the study was to investigate whether there were differences in the predictors of dental services utilisation between rural and urban populations in Brazil.

## Methods

### Study population

The study was conducted using data from the National Health Survey (NHS), a nationwide household-based health survey conducted in Brazil in 2013. The NHS aimed to characterise the health status of the Brazilian population and obtain information on health-related behaviours, and health services utilisation [25].

The study population consisted of 60,202 residents from the households that participated in the NHS. In the first sampling stage, the census tracts or their clusters were selected using probability-proportional-to-size samples. The households in each primary sampling unit were selected through simple random sampling in the second stage. One adult aged 18 years or older in each household was randomly selected to complete the oral health questionnaire, corresponding to the third stage of sample selection. The sample of the NHS was representative for Brazil, geographic regions, states, metropolitan regions of the state capitals, and state capitals [25].

### Outcome variables

**Fig. 1:**
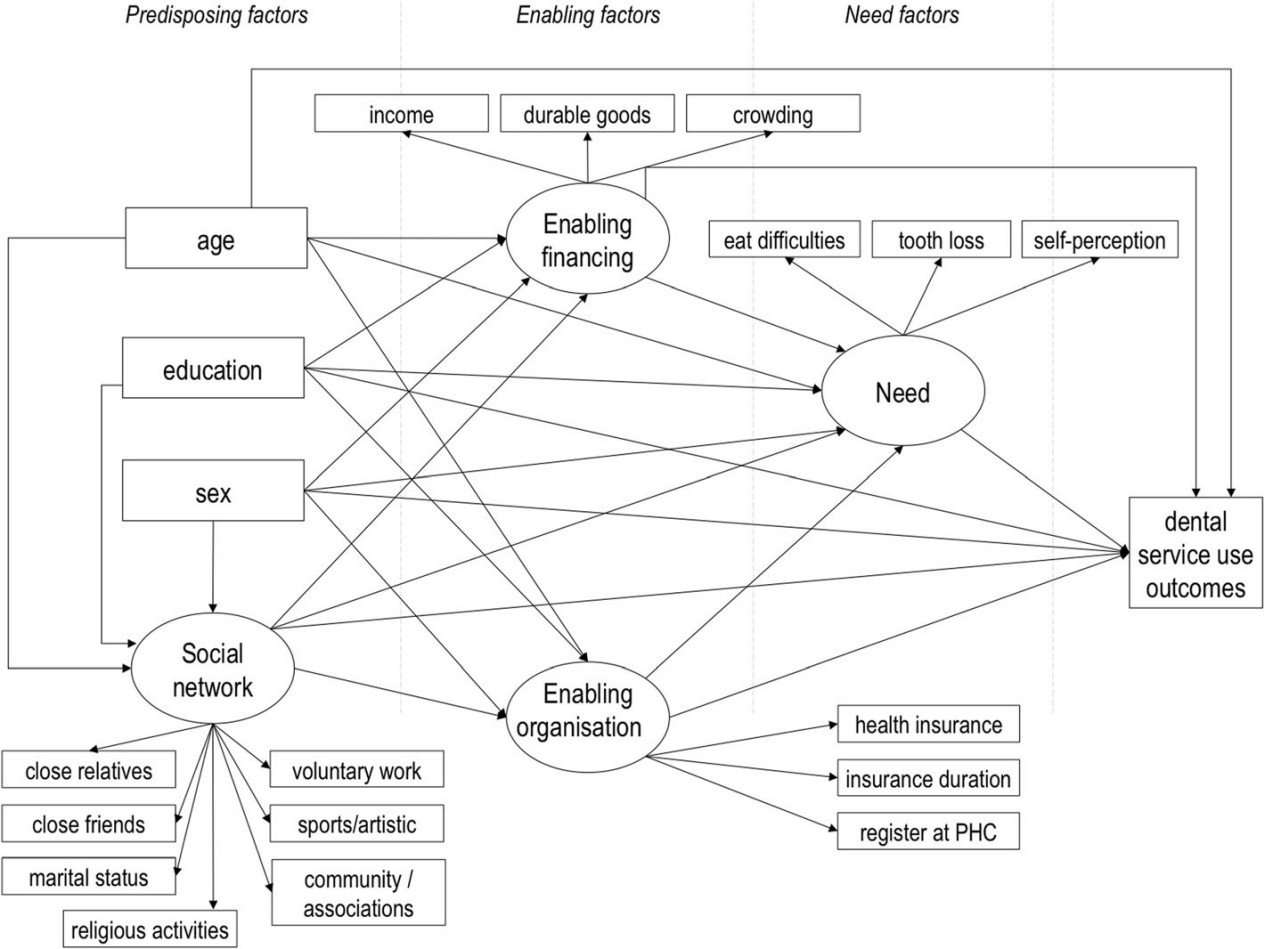
Hypothesised theoretical model for dental services utilisation in Brazilian adults using the Andersen behavioural model. Arrows indicate hypothesised direct relationships between variables. Measured variables are in rectangles and latent variables in ellipses

The two outcome variables were non-utilisation of dental services (0 = at least one dental visit throughout life, 1 = never been to the dentist), and the interval since the last dental visit. In the latter, participants who had already had a dental visit were classified as 1: < 1 year, 2: 1 to < 2 years, 3: 2 to < 3 years, and 4: ≥ 3 years. Figure 1 shows the theoretical model grouping the independent variables as predisposing, enabling and need factors based upon the Andersen behavioural model [24].

### Predictor variables

Predisposing factors included three observed variables (inside rectangles) and one latent variable (inside circle). Age and sex (0 = male, 1 = female) were predisposing demographics. Education was the observed predisposing social variable assessed using complete years of education. Social network was a latent predisposing social variable based on Berkman and Syme social network index modified by Loucks et al. [26]. Seven indicators were used to assess social network: (1) living with spouse or partner (0 = no, 1 = yes); (2) number of close friends; (3) number of close relatives; (4) participation in sports or artistic group activities; (5) participation in community meetings and associations; (6) participation in religious meetings; (7) voluntary services. Indicators 4, 5, 6 and 7 were related to the last 12 months according to the following options: 1 = more than once a week, 2 = once a week, 3 = two to three times a month, 4 = once a year, 5 = sometimes in the year, 6 = never.

Enabling factors were two latent variables: financing and organisation. Enabling financing was measured using three indicators: personal income, number of durable goods in the household and household crowding. Personal income was the sum of the monthly gross income of the individual. Number of durable goods was calculated based on the Brazilian economic classification criteria proposed in 2015 [27]. A final score was obtained by adding up the scores of each durable good. Household crowding was the ratio between the number of residents in the household and the number of rooms, excluding bathrooms, the kitchen and garage. Enabling organisation factors are facilitating aspects related to a regular source of health care services [24]. Enabling organisation was represented by three variables: having private health insurance (0 = no, 1 = yes), duration of private health insurance (1 = none, 2 = ≤ 6 months, 3 = > 6 to 12 months, 4 = > 12 to 24 months, 4 = > 24 months) and registration in primary health care (PHC) (1 = no/do not know, 2 = yes).

Need factors were represented by a latent variable measured by three indicators: self-reported tooth loss (ranging from 0 = no tooth lost to 32 = lost all teeth), self-rated oral health (1 = very good, 2 = good, 3 = regular, 4 = poor, 5 = very poor) and eating difficulties due to oral problems (1 = none, 2 = slight, 3 = regular, 4 = intense, 5 = very intense).

### Analytic plan

The variables were described through proportions and respective 95% confidence intervals (95%CI), considering the complex sample design and the sampling weights. The hypothesised model was examined through SEM using the AMOS software (version 24.0, SPSS, IBM, Meadville, PA). Asymptotic distribution free (ADF) bootstrap method with 900 replications was used to estimate parameters, considering the large sample size and the no need for distributional assumptions. Missing data for income variable (*n* = 9151) was addressed with Bayesian multiple data imputation (*n* = 10 datasets). In the analyses, a two-step strategy was adopted. Initially, the measurement model was tested using confirmatory factor analysis (CFA) to test the indicators (observed variables) of the latent variables according to the factorial loadings and statistical significance. Then, structural models for the whole sample and according to rural and urban areas were assessed using SEM to estimate the direct, indirect, and total effects, with 95% bias-corrected CI. The adequacy of the model fit was evaluated using the following criteria GFI, CFI and TLI ≥ 0.95, RMSEA ≤0.05 (90%CI upper limit ≤0.08) and SRMR ≤0.08.

Finally, multi-group analyses were performed to evaluate the configural and structural invariance of the models between urban (*n* = 49,245) and rural (*n* = 10,957) areas. Models were re-estimated for urban and rural areas separately by removing non-significant direct paths according to the theoretical model to obtain a parsimonious model. Standardised and non-standardised coefficients (β) and their 95% bias-corrected CIs were estimated for each group (urban and rural) to allow comparisons within and between the models [28].

## Results

Data describing the study populations in the rural and urban areas are summarised in Table 1. The proportion of adults who have never visited a dentist was greater in the rural than in the urban area. Moreover, having visited a dentist within the last 12 months was more common among individuals living in urban areas.

**Table 1:** Utilisation of dental services, predisposing, enabling and need characteristics of the study population, Brazil, 2013

Variables	Brazil (***n*** = 60,202)	Rural (***n*** = 10,957)	Urban (***n*** = 49,245)
Utilisation of dental services (%, 95CI)			
Never been to the dentist	3.3 (3.1–3.6)	8.6 (7.7–9.7)	2.5 (2.2–2.8)
≥ 3 years since last dental visit	24.1 (23.4–24.7)	34.5 (32.7–36.3)	22.4 (21.7–23.1)
2 to < 3 years since last dental visit	8.9 (8.5–9.3)	9.4 (8.5–10.3)	8.8 (8.4–9.3)
1 to < 2 years since last dental visit	19.3 (18.7–19.8)	16.3 (15.2–17.5)	19.7 (19.1–20.4)
< 1 year since last dental visit	44.4 (43.6–45.2)	31.1 (29.5–32.9)	46.6 (45.7–47.4)
*Predisposing variables*			
Age (mean, 95%CI)	42.9 (42.9–43.0)	43.4 (42.9–44.0)	42.8 (42.7–43.0)
Sex (%, 95CI)			
Male	47.1 (46.4–47.8)	51.3 (49.7–52.8)	46.4 (46.2–46.7)
Female	52.9 (52.2–53.6)	48.7 (47.2–50.3)	53.6 (53.3–53.8)
Education - years of study (mean, 95%CI)	8.2 (8.1–8.3)	5.1 (5.0–5.3)	8.7 (8.6–8.8)
Lives with spouse or partner (%, 95CI)			
Yes	61.2 (60.5–61.9)	70.1 (68.6–71.6)	59.8 (59.0–60.5)
Number of close relatives (mean, 95%CI)	3.0 (2.9–3.0)	3.3 (3.1–3.5)	2.9 (2.9–3.0)
Number of close friends (mean, 95%CI)	2.2 (2.1–2.2)	2.4 (2.2–2.6)	2.1 (2.1–2.2)
Participation in sports or artistic group activities (%, 95CI)			
More than once a week	10.6 (10.1–11.1)	5.7 (4.9–6.6)	11.4 (10.8–11.9)
Once a week	6.2 (5.9–6.6)	5.2 (4.5–5.9)	6.4 (6.0–6.8)
2–3 times a month	3.0 (2.7–3.2)	2.2 (1.8–2.7)	3.1 (2.8–3.4)
Sometimes in the year	4.9 (4.6–5.3)	4.1 (3.5–4.8)	5.1 (4.7–5.4)
Once a year	1.4 (1.3–1.6)	1.5 (1.1–2.0)	1.4 (1.3–1.6)
Not once in the year	73.8 (73.0–74.6)	81.3 (79.8–82.7)	72.6 (71.7–73.5)
Participation in community meetings and associations (%, 95CI)			
More than once a week	1.1 (1.0–1.3)	0.6 (0.4–0.9)	1.2 (1.1–1.4)
Once a week	1.6 (1.4–1.8)	1.3 (1.0–1.7)	1.6 (1.5–1.8)
2 to 3 times a month	2.3 (2.1–2.5)	3.3 (2.7–4.0)	2.2 (2.0–2.4)
Sometimes in the year	8.4 (8.0–8.8)	14.5 (13.1–16.0)	7.4 (7.0–7.9)
Once a year	2.7 (2.4–2.9)	3.9 (3.2–4.6)	2.5 (2.3–2.7)
Not once in the year	83.9 (83.3–84.5)	76.4 (74.7–78.1)	85.1 (84.4–85.7)
Participation in voluntary services (unpaid) (%, 95CI)			
More than once a week	1.5 (1.3–1.6)	0.4 (0.3–0.6)	1.6 (1.4–1.8)
Once a week	1.9 (1.7–2.1)	1.2 (0.9–1.6)	2.0 (1.8–2.3)
2–3 times a month	1.4 (1.3–1.6)	1.1 (0.9–1.5)	1.5 (1.3–1.7)
Sometimes in the year	5.3 (4.9–5.6)	5.5 (4.7–6.5)	5.2 (4.8–5.6)
Once a year	2.1 (1.8–2.3)	2.4 (1.9–2.9)	2.0 (1.8–2.2)
Not once in the year	87.9 (87.3–88.4)	89.3 (88.1–90.4)	87.6 (87.0–88.2)
Participation in religious meetings (%, 95CI)			
More than once a week	15.3 (14.8–15.9)	9.3 (8.4–10.2)	16.3 (15.7–17.0)
Once a week	21.4 (20.8–22.0)	17.2 (15.9–18.7)	22.1 (21.4–22.8)
2–3 times a month	11.1 (10.7–11.5)	13.7 (12.7–14.8)	10.6 (10.2–11.1)
Sometimes in the year	18.7 (18.1–19.3)	29.5 (27.7–31.3)	17.0 (16.3–17.6)
Once a year	3.6 (3.3–3.8)	5.9 (5.2–6.6)	3.2 (2.9–3.5)
Not once in the year	30.0 (29.2–30.7)	24.5 (23.1–25.8)	30.8 (30.0–31.7)
*Enabling variables*			
Personal income (BRL, mean, 95%CI)	1686.6 (1618.7–1754.4)	827.0 (795.7–858.3)	1819.1 (1740.8–1897.4)
Number of residents in the household (mean, 95%CI)	3.5 (3.5–3.6)	3.8 (3.7–3.8)	3.5 (3.5–3.5)
Number of rooms in the household (mean, 95%CI)	6.2 (6.1–6.2)	5.8 (5.7–5.9)	6.3 (6.2–6.3)
Durable goods and items (mean, 95%CI)	14.4 (14.2–14.6)	8.6 (8.4–8.8)	15.3 (15.1–15.6)
Health insurance (%, 95CI)			
Yes	30.3 (29.4–31.2)	6.9 (5.9–8.0)	34.0 (33.0–35.0)
Duration of health insurance (%, 95CI)			
None	69.7 (68.8–70.6)	93.1 (92.0–94.1)	66.0 (64.0–67.0)
Up to 6 months	2.2 (2.0–2.4)	0.5 (3.5–7.6)	2.5 (2.2–2.7)
More than 6 months to 1 year	1.8 (1.6–2.0)	0.4 (0.3–0.6)	2.0 (1.8–2.2)
More than 1 year to 2 year2	3.0 (2.7–3.3)	0.8 (0.6–1.1)	3.3 (3.0–3.6)
More than 2 years	23.3 (22.5–24.1)	5.1 (4.2–6.2)	26.2 (25.3–27.1)
Registration in the primary care (%, 95CI)			
Yes	54.6 (53.4–55.8)	72.1 (69.5–74.5)	51.8 (50.4–53.2)
No	34.6 (33.5–35.7)	17.0 (15.4–18.7)	37.4 (36.1–38.7)
Did not know	10.8 (10.2–11.4)	10.9 (9.2–12.9)	10.8 (10.1–11.4)
*Need-related variables*			
Self-rated oral health (%, 95CI)			
Very good	9.9 (9.3–10.4)	4.2 (3.5–5.0)	10.8 (10.2–11.4)
Good	57.6 (56.8–58.3)	52.4 (50.9–53.8)	58.4 (57.6–59.2)
Regular	26.7 (26.0–27.3)	34.5 (32.9–36.1)	25.4 (24.7–26.2)
Poor	4.9 (4.6–5.2)	7.6 (6.8–8.4)	4.5 (4.2–4.9)
Very poor	0.9 (0.8–1.1)	1.3 (1.0–1.7)	0.9 (0.8–1.0)
Eating difficulties due to oral problems (%, 95CI)			
None	89.6 (89.1–90.0)	83.7 (82.4–84.5)	90.5 (90.0–90.9)
Slight	5.3 (5.0–5.6)	8.0 (7.2–8.9)	4.8 (4.5–5.2)
Regular	3.6 (3.4–3.9)	5.9 (5.2–6.8)	3.2 (3.0–3.5)
Intense	1.2 (1.1–1.4)	2.0 (1.6–2.6)	1.1 (1.0–1.3)
Very intense	0.3 (0.2–0.4)	0.3 (0.2–0.4)	0.3 (0.2–0.4)
Self-reported tooth loss (mean, 95%CI)	8.0 (7.9–8.1)	10.8 (10.4–11.2)	7.6 (7.4–7.7)

**Fig. 2:**
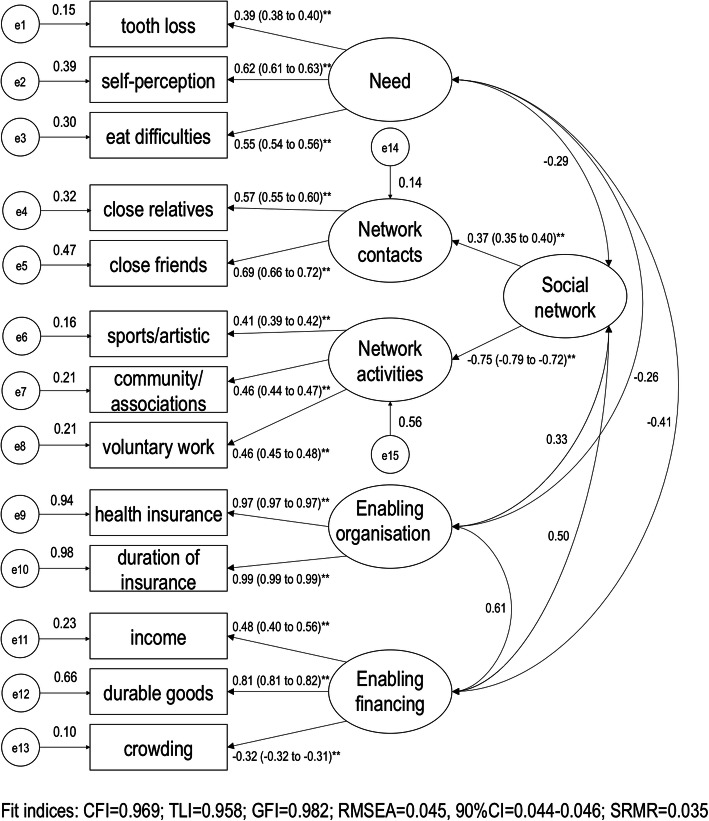
Confirmatory factor analysis of the 4-factors 13 items (measurement model). Obtained through bootstrapped ADF item loadings (standardised coefficients / bias-corrected 95% confidence intervals). ^**^Significant standardised coefficients (*P* < 0.01)

Confirmatory factor analysis (CFA) supported the use of the four latent variables in the measurement model (Fig. 2), with a good model fit. Social network was a two-order latent variable to reduce the correlations between errors and improve the reliability of the indicators. The observed variables “participation in religious activities” (loading coefficient = 0.272) and “marital status” (loading coefficient = − 0.008) were removed from the measurement model because the factorial loadings were lower than 0.30. The correlation coefficient between the two social network latent variables in the first-order CFA was − 0.26, demonstrating good discriminant validity. In the second-order analysis, coefficient correlations between the four latent social network variables ranged from − 0.41 to 0.61. Registration in PHC (loading coefficient = − 0.181) was removed from the enabling organisation latent variable and was retained as an observed variable in the structural models.

**Fig. 3:**
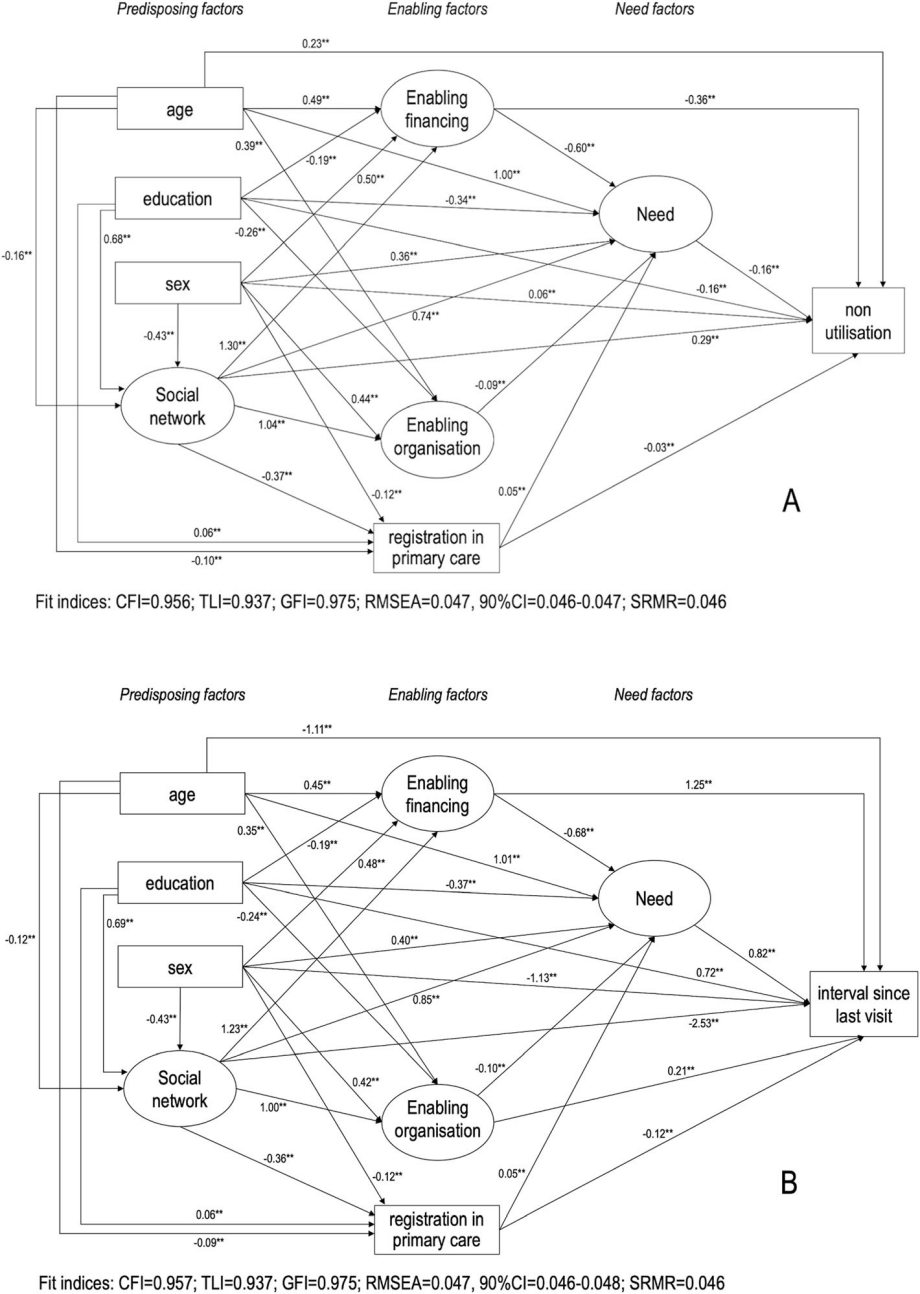
Parsimonious models of associations between predisposing, enabling and need factors and dental services utilisation outcomes. A, non-utilisation of dental services, and B, interval since the last dental visit. Solid lines indicate standardised direct effects. ** *P* < 0.01

Parsimonious models for non-utilisation of dental services and interval since last dental visit for all participants are shown in Fig. 3. In the former model of the non-utilisation outcome, the direct path from enabling organisation factor to non-utilisation of dental services was non-significant and thus removed. For this outcome, the full and parsimonious models were not statistically different (Chi-square difference = 0.001, *P* = 0.975). For the interval since last dental attendance outcome, all the direct paths in the full model were statistically significant, and the full specified model was maintained. The detailed direct and indirect estimates for non-utilisation of dental services and interval since last dental visit are shown in the Additional file 1 and Additional file 2, respectively. The full and parsimonious models showed acceptable fit to the data.

Multi-group analyses were performed once the models involving all participants were adjusted. The initial analysis showed that the proposed measurement model presented acceptable fit to the data, simultaneously, among individuals living in the urban and rural areas, demonstrating the configural invariance of the model (GFI = 0.980; CFI = 0.964; TLI = 0.951; RMSEA = 0.034, 90%CI = 0.033–0.034; SRMR = 0.038). Thus, the factorial arrangement was similar in both groups.

**Fig. 4:**
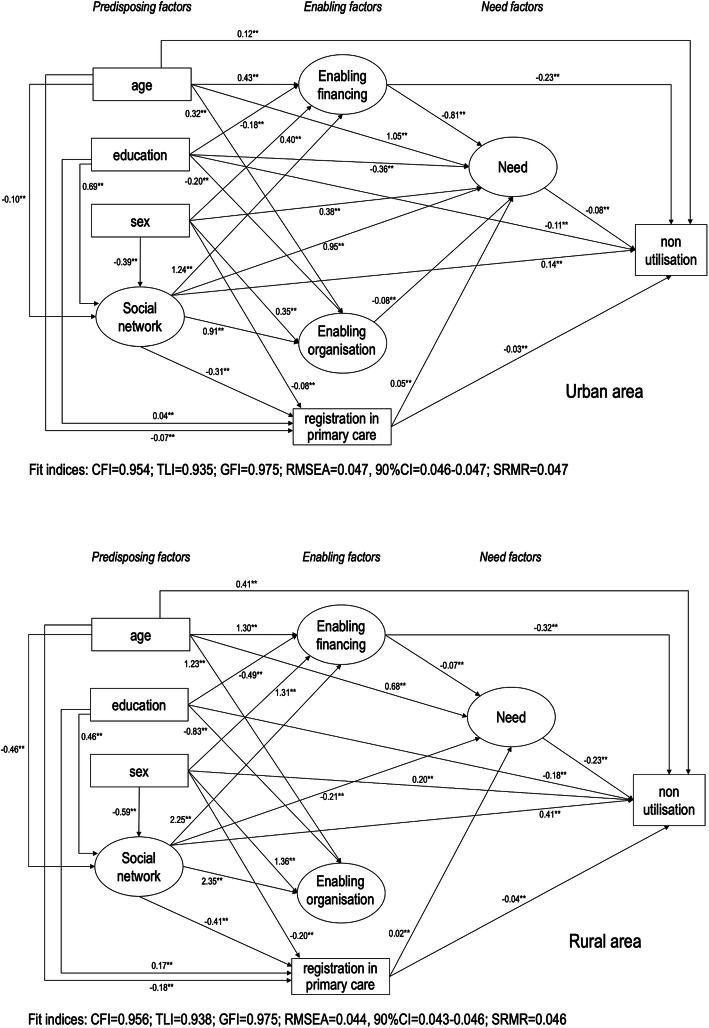
Parsimonious models for non-utilisation of dental services in urban and rural areas. Solid lines indicate standardised direct effects. ** *P* < 0.01

Analysis of structural invariance for non-utilisation of dental services model revealed that relationships between variables differed between rural and urban areas (constrained vs. unconstrained unstandardised regression weights were different, *P* < 0.01). The parsimonious models showed acceptable fits to the data. The models are shown in Fig. 4 and the standardised estimates are described in the supplementary material [see Additional file 1]. The greater standardised total effects of non-utilisation of dental services in the urban population were lower enabling financing (β = − 0.17), education (β = − 0.14) and social network (β = − 0.13). Lower enabling financing (β = − 0.30), perceived oral health need (β = − 0.23) and social network (β = − 0.21) were the main total effects in the rural population.

**Fig. 5:**
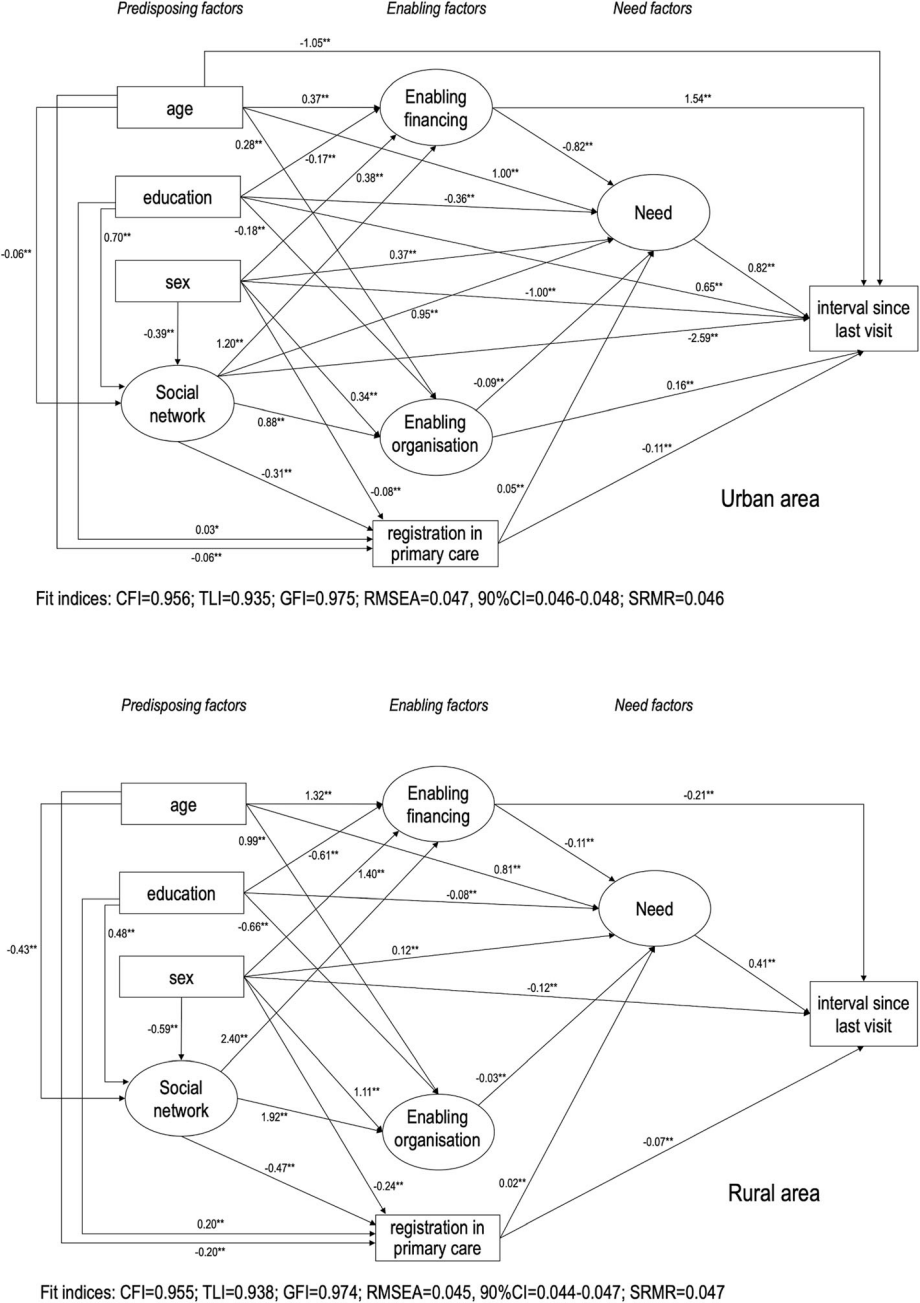
Parsimonious models for the interval since the last dental visit in urban and rural areas. Solid lines indicate standardised direct effects. * *P* < 0.05, ** *P* < 0.01

The analysis of structural invariance for the interval since the last dental visit also demonstrated that the relationships between variables were different between urban and rural areas. The evaluation of the re-estimated models in the urban population showed that the model does not undergo structural alteration and the goodness of fit remained adequate. For the rural areas, the direct path between social network and need was non-significant, as well as the direct path from schooling, age, social network and enabling organisation to interval since last dental visit (Fig. 5). The greater standardised total effects of interval since last dental visit (the longest time) in the urban population were better enabling financing (β = 0.87), perceived oral health needs (β = 0.82) and lower social networks (β = − 0.68). Worse social networks (β = − 0.62), better perceived oral health needs (β = 0.41), lower enabling financing (β = − 0.26) and older age (β = 0.26) represented the main total effects in the rural population (see Additional file 2).

The standardised coefficients of all indirect paths for non-utilisation of dental services and interval since the last dental visit are available in Additional file 3 and Additional file 4. Non-standardised estimates of the models are shown in Additional file 5 and Additional file 6.

## Discussion

Our findings showed that adults living in rural areas used dental services less frequently than those living in urban areas. In addition, among those who have used dental services, interval since the last dental visit was longer among residents in the rural areas than those in the urban ones. These findings reinforce the persistence of inequalities in the use of dental services between rural and urban areas among adults and elderly people observed in previous surveys conducted in Brazil [13, 14, 16].

Enabling financing presented the highest total effect on non-utilisation of dental services in both geographical contexts. This finding was similar for interval since the last dental visit in the urban population. This suggests that the higher the financial capacity, the easier it will be to overcome barriers to access dental services [5, 29]. This effect was greater among people living in rural areas. The association (direct and total effects) between enabling financing and interval since the last dental visit was opposite between rural and urban areas. While better financial conditions predicted a higher frequency of dental utilisation in the rural areas, greater financial conditions were associated with a longer interval since the last dental visit in the urban areas. This paradox was not explained by fewer oral health needs in adults from urban areas [11]. People living in rural areas usually have poor financial conditions and may face more difficulties in accessing services due to long distances between their households and oral health care units. In this population, greater material resources may facilitate access to dental services [4, 30].

Private health insurance was not associated with the non-utilisation of dental services in the rural population. However, health insurance was indirectly linked to the utilisation of dental services in urban areas mediated by need factors. The low availability of dental services in rural areas and the low coverage of dental care within private health insurance companies in Brazil may explain this finding. Health insurance was also linked to a longer interval since the last dental visit in the urban areas. Information whether dental care is included in the health insurance was not considered by the NHS. Indeed, having health insurance seems to indicate a better socioeconomic status [14].

Registration in PHC was a meaningful factor related to utilisation of dental services in the rural and urban areas since adults registered in PHC were more likely to have recently seen a dentist than those who were not registered in PHC. Regular access to dental care is an important determinant of dental service utilisation [31], which is also related to preventive dental healthcare in both urban and rural areas [32].

Perceived oral health need demonstrated to have a relevant role in the use of dental services in the rural population. On the other hand, social networks and schooling presented greater effects in the use of dental services in urban areas. In this population, those in greater need were more likely to have recently seen a dentist. A previous study involving adult rural workers reported that individuals with more oral symptoms were less likely to have a dental visit over the last year [31]. These findings suggest that oral health care in rural areas in Brazil is more focused on dental treatment rather than on prevention. Dental extractions are more common in rural populations than in the urban ones according to previous research [33]. Therefore, enhancing oral health promotion and preventive dental care in rural areas seem paramount to tackle dental care inequalities [6, 30, 32, 34]. Also, a greater interaction and cooperation between health professionals and residents may be necessary for social transformation [35, 36].

Schooling was associated with a shorter dental visit interval. Although this has been previously reported [4, 29, 37], this study revealed interesting pathways by which education may influence dental attendance since social networks mediated the aforementioned relationship in rural areas. On the other hand, need, enabling financing and social networks were the main mediators between education and dental attendance in the urban settings. Our findings also suggest that individuals with higher education also showed stronger social ties, greater financial resources, and better oral health.

Women and older adults reported greater dental attendance than men and younger people. The direct effects indicated that the higher the age predicted the lower was the dental attendance. However, total effects showed that the likelihood of having used the service increased with age. This effect was due to indirect paths through need and financial factors. Notwithstanding, the last dental visit was longer among older individuals than younger ones, mainly in rural areas. Non-standardised effects of sex on dental services utilisation were greater in rural than in urban areas, showing that men use dental services less frequently. This relationship occurred through different mechanisms in rural and urban areas, including via need, social networks and financial enabling pathways. The demographic disparities in the utilisation of dental services revealed that men and older adults deserve further attention from policymakers and stakeholders to reduce dental services use inequalities. Improving access to dental care for these population groups would also require better healthcare organisation at a local level to deliver health services that can meet their needs [12]. Besides the difficulties in accessing dental services by older adults [12, 29], the higher rates of edentulism is this age group may explain the non-recent use of dental services since elders tend to believe that regular dental visits are only necessary for those with natural teeth [16].

The present study supports the use of the Andersen behavioural model as a robust theoretical model to investigate the determinants of dental services utilisation [21, 23]. The behavioural model has been used to assess health services utilisation, including oral health services [21, 38]. The identification of relevant predictors of dental services utilisation in urban and rural areas in this study enhances the understanding of the possible barriers of dental services utilisation. Ultimately, our findings might support the development of public policies to increase equitable access to dental care and consequently reduce oral health iniquities [21, 23, 24, 39].

### Limitations

Causal inferences should be cautious due to the cross-sectional design of the study. The use of a robust framework underlying the theoretical relationships among variables is a way to minimise this limitation. The large sample size contributed to obtaining estimates with adequate precision. However, the remarkable heterogeneity of dental services utilisation across Brazilian regions and cities would require regional studies to address local specificities. Another limitation of this study was the lack of information regarding the reason for the last dental attendance and therefore this aspect was not assessed. The rural and urban classification of the population in this study followed the administrative division of the cities according to the Brazilian legislation. Yet, this categorisation does not consider the characteristics of urbanisation (e.g. population size and density, services availability), which may have introduced some classification bias [12].

## Conclusions

This study found that dental services utilisation was less frequent in rural areas. The theoretical model was supported by empirical data and showed different relationships between the predictors of dental services utilisation in the two evaluated contexts. Financial limitations were a relevant aspect of dental services utilisation in rural areas. Dental services availability through primary care was associated with dental services utilisation in both contexts. Nonetheless, the greater effect was observed in rural areas. A higher level of education also predicted the utilisation of dental services through indirect pathways. Dental services utilisation was lower among males, and a higher age was related to longer interval since the last dental visit, with greater effects in the rural areas.

## Supplementary information

**Additional file 1.** Direct, indirect and total standardised effects on the structural equation model for the non-utilisation of dental services in rural and urban contexts.

**Additional file 2.** Direct, indirect and total standardised effects on the structural equation model for the interval since the last dental visit outcome in rural and urban contexts.

**Additional file 3.** Calculation of specific indirect paths of total indirect effects for the non-utilisation of dental services.

**Additional file 4.** Calculation of specific indirect paths of total indirect effects for the interval since the last dental visit.

**Additional file 5.** Direct, indirect and total non-standardised effects on the structural equation model for the non-utilisation of dental services in rural and urban contexts.

**Additional file 6.** Direct, indirect and total non-standardised effects on the structural equation model for the interval since the last dental visit outcome in rural and urban contexts.

## Data Availability

The National Health Survey data analysed during the current study is in the public domain, available in the Instituto Brasileiro de Geografia e Estatística (IBGE) repository (ftp://ftp.ibge.gov.br/PNS/2013/microdados/pns_2013_microdados_2017_03_23.zip).

## Abbreviations

ADF: asymptotic distribution free
CFA: confirmatory factor analysis
NHS: National Health Survey
PHC: primary health care
SEM: structural equation modeling

## References

[1] Garcia-Subirats I, Vargas Lorenzo I, Mogollon-Perez AS, De Paepe P, Silva MR, Unger JP (2014). Determinants of the use of different healthcare levels in the general system of social security in health in Colombia and the unified health system in Brazil. Gac Sanit.

[2] Sibley LM, Weiner JP (2011). An evaluation of access to health care services along the rural-urban continuum in Canada. BMC Health Serv Res.

[3] Montgomery AL, Ram U, Kumar R, Jha P (2014). Maternal mortality in India: causes and healthcare service use based on a nationally representative survey. PLoS One.

[4] Allison RA, Manski RJ (2007). The supply of dentists and access to care in rural Kansas. J Rural Health.

[5] Curtis B, Evans RW, Sbaraini A, Schwarz E (2007). Geographic location and indirect costs as a barrier to dental treatment: a patient perspective. Aust Dent J.

[6] Barnett T, Hoang H, Stuart J, Crocombe L (2016). "sorry, I'm not a dentist": perspectives of rural GPs on oral health in the bush. Med J Aust.

[7] Gaber A, Galarneau C, Feine JS, Emami E (2018). Rural-urban disparity in oral health-related quality of life. Community Dent Oral Epidemiol.

[8] Heaton LJ, Smith TA, Raybould TP (2004). Factors influencing use of dental services in rural and urban communities: considerations for practitioners in underserved areas. J Dent Educ.

[9] De Marchi RJ, Leal AF, Padilha DM, Brondani MA (2012). Vulnerability and the psychosocial aspects of tooth loss in old age: a southern Brazilian study. J Cross Cult Gerontol.

[10] Pucca GA, Gabriel M, Araujo ME, Almeida FC (2015). Ten years of a National Oral Health Policy in Brazil: innovation, boldness, and numerous challenges. J Dent Res.

[11] Antunes JL, Peres MA, Jahn GMJ, Levy BBS (2006). The use of dental care facilities and oral health: a multilevel approach of schoolchildren in the Brazilian context. Oral Health Prev Dent.

[12] Antunes JL, Peres MA, de Campos Mello TR, Waldman EA (2006). Multilevel assessment of determinants of dental caries experience in Brazil. Community Dent Oral Epidemiol.

[13] Travassos C, Viacava F (2007). Acesso e uso de serviços de saúde em idosos residentes em áreas rurais, Brasil, 1998 e 2003. Cad Saude Publica..

[14] Macinko J, Lima Costa MF (2012). Access to, use of and satisfaction with health services among adults enrolled in Brazil's family health strategy: evidence from the 2008 National Household Survey. Tropical Med Int Health.

[15] Victora CG, Vaughan JP, Barros FC, Silva AC, Tomasi E (2000). Explaining trends in inequities: evidence from Brazilian child health studies. Lancet..

[16] Martins AM, Barreto SM, Pordeus IA (2008). Characteristics associated with use of dental services by dentate and edentulous elders: the SB Brazil project. Cad Saude Publica.

[17] Silva AE, Langlois CO, Feldens CA (2013). Use of dental services and associated factors among elderly in southern Brazil. Rev Bras Epidemiol.

[18] Gomes AM, Thomaz EB, Alves MT, Silva AA, Silva RA. Fatores associados ao uso dos serviços de saúde bucal: estudo de base populacional em municípios do Maranhão, Brasil Cien Saude Colet 2014;19:629–640. 10.1590/1413-81232014192.23252012 24863839

[19] Pinto RS, Abreu MH, Vargas AM (2014). Comparing adult users of public and private dental services in the state of Minas Gerais, Brazil. BMC Oral Health.

[20] Pescosolido BA, Kronenfeld JJ. Health, illness, and healing in an uncertain era: challenges from and for medical sociology. J Health Soc Behav. 1995;SpecNo:5–33. 7560849

[21] Aday LA, Andersen RM (2005). Health care utilization and behavior, Models of *Encyclopedia of Biostatistics*.

[22] Marshman Z, Porritt J, Dyer T, Wyborn C, Godson J, Baker S (2012). What influences the use of dental services by adults in the UK?. Community Dent Oral Epidemiol.

[23] Baker SR (2009). Applying Andersen's behavioural model to oral health: what are the contextual factors shaping perceived oral health outcomes?. Community Dent Oral Epidemiol.

[24] Andersen RM, Davidson PL, Kominski GF (2014). Improving access to care in America: individual and contextual indicators. Changing the U.S. health care system: key issues in health services policy and management.

[25] Szwarcwald CL, Malta DC, Pereira CA, Vieira ML, Conde WL, Souza Júnior PR (2014). Pesquisa Nacional de Saúde no Brasil: concepção e metodologia de aplicação. Cien Saude Colet.

[26] Loucks EB, Sullivan LM, D’Agostino RB, Larson MG, Berkman LF, Benjamin EJ (2006). Social networks and inflammatory markers in the Framingham heart study. J Biosoc Sci.

[27] Kamakura WA, Mazzon JA (2013). Socioeconomic status and consumption in an emerging economy. IJRM..

[28] Kline RB (2015). Principles and practice of structural equation modeling.

[29] Afonso-Souza G, Nadanovsky P, Chor D, Faerstein E, Werneck GL, Lopes CS (2007). Association between routine visits for dental checkup and self-perceived oral health in an adult population in Rio de Janeiro: the pro-Saude study. Community Dent Oral Epidemiol.

[30] Barnett T, Hoang H, Stuart J, Crocombe L (2015). Non-dental primary care providers' views on challenges in providing oral health services and strategies to improve oral health in Australian rural and remote communities: a qualitative study. BMJ Open.

[31] Finlayson TL, Gansky SA, Shain SG, Weintraub JA (2010). Dental utilization among Hispanic adults in agricultural worker families in California's Central Valley. J Public Health Dent.

[32] Khan A, Thapa JR, Zhang D (2017). Preventive dental checkups and their association with access to usual source of care among rural and urban adult residents. J Rural Health.

[33] Fischer TK, Peres KG, Kupek E, Peres MA (2010). Primary dental care indicators: association with socioeconomic status, dental care, water fluoridation and family health program in southern Brazil. Rev Bras Epidemiol..

[34] Guo Y, Logan HL, Dodd VJ, Muller KE, Marks JG, Riley JL (2014). Health literacy: a pathway to better oral health. Am J Public Health.

[35] Taylor J, Carlisle K, Farmer J, Larkins S, Dickson-Swift V, Kenny A (2018). Implementation of oral health initiatives by Australian rural communities: factors for success. Health Soc Care Community.

[36] Barnett T, Hoang H, Stuart J, Crocombe L (2017). The relationship of primary care providers to dental practitioners in rural and remote Australia. BMC Health Serv Res.

[37] Reda SM, Krois J, Reda SF, Thomson WM, Schwendicke F (2018). The impact of demographic, health-related and social factors on dental services utilization: systematic review and meta-analysis. J Dent.

[38] Ricketts TC, Goldsmith LJ (2005). Access in health services research: the battle of the frameworks. Nurs Outlook.

[39] Babitsch B, Gohl D, von Lengerke T. Re-revisiting Andersen's Behavioral Model of Health Services Use: a systematic review of studies from 1998–2011. Psychosoc Med. 2012;9:Doc11. 10.3205/psm000089 PMC3488807 23133505

