# Supplementary material for: Utilisation of dental services by Brazilian adults in rural and urban areas: a multi-group structural equation analysis using the Andersen behavioural model
Source: BMC Public Health. 2020 Jun 17;20:953. doi: 10.1186/s12889-020-09100-x (PMC7301519; doi:10.1186/s12889-020-09100-x)
Supplement: Supplementary file 1 — Additional file 1. Direct, indirect and total standardised effects on the structural equation model for the non-utilisation of dental services in rural and urban contexts. [file 12889_2020_9100_MOESM1_ESM.docx]

Additional file 1. Direct, indirect and total standardised effects on the structural equation model for the non-utilisation of dental services in rural and urban contexts.

| **Parameter** | **Rural** | | **Urban** | | **Total sample** | |
| --- | --- | --- | --- | --- | --- | --- |
|  | **β** | **Bias-corrected**  **95% CI** | **β** | **Bias-corrected**  **95% CI** | **β** | **Bias-corrected**  **95% CI** |
| **Direct effects** |  |  |  |  |  |  |
| Need → no use | -0.23 | -0.32 to -0.17 ^**^ | -0.08 | -0.11 to -0.05 ^**^ | -0.16 | -0.22 to -0.13 ^**^ |
| Social network → no use | 0.41 | 0.21 to 0.95 ^**^ | 0.14 | 0.11 to 0.17 ^**^ | 0.29 | 0.18 to 0.48 ^**^ |
| Social network → need | -0.21 | -0.23 to -0.18 ^**^ | 0.95 | 0.53 to 1.57 ^**^ | 0.74 | 0.44 to 1.13 ^**^ |
| Social network → enabling financing | 2.25 | 1.61 to 3.66 ^**^ | 1.24 | 1.17 to 1.31 ^**^ | 1.30 | 1.23 to 1.39 ^**^ |
| Social network → enabling organisation | 2.35 | 1.62 to 3.80 ^**^ | 0.91 | 0.86 to 0.98 ^**^ | 1.04 | 0.97 to 1.11 ^**^ |
| Social network → registration in primary care | -0.41 | -0.70 to -0.25 ^**^ | -0.31 | -0.34 to -0.28 ^**^ | -0.37 | -0.40 to -0.34 ^**^ |
| Enabling financing → no use | -0.32 | -0.43 to -0.26 ^**^ | -0.23 | -0.26 to -0.20 ^**^ | -0.36 | -0.50 to -0.29 ^**^ |
| Enabling financing → need | -0.07 | -0.10 to 0.04 ^**^ | -0.81 | -1.24 to -0.53 ^**^ | -0.60 | -0.84 to -0.44 ^**^ |
| Enabling organisation → need | - | - | -0.08 | -0.11 to -0.06 ^**^ | -0.09 | -0.12 to -0.07 ^**^ |
| Registration in primary care → no use | -0.04 | -0.06 to -0.02 ^**^ | -0.03 | -0.04 to -0.02 ^**^ | -0.03 | -0.04 to -0.02 ^**^ |
| Registration in primary care → need | 0.02 | 0.01 to 0.04 ^**^ | 0.05 | 0.04 to 0.06 ^**^ | 0.05 | 0.04 to 0.06 ^**^ |
| Education → no use | -0.18 | -0.36 to -0.11 ^**^ | -0.11 | -0.13 to -0.09 ^**^ | -0.16 | -0.21 to -0.13 ^**^ |
| Education → need | - | - | -0.36 | -0.50 to -0.27 ^**^ | -0.34 | -0.45 to -0.25 ^**^ |
| Education → social network | 0.46 | 0.40 to 0.52 ^**^ | 0.70 | 0.67 to 0.72 ^**^ | 0.68 | 0.66 to 0.70 ^**^ |
| Education → enabling financing | -0.49 | -1.12 to -0.19 ^**^ | -0.18 | -0.25 to -0.11 ^**^ | -0.19 | -0.27 to -0.13 ^**^ |
| Education → enabling organisation | -0.83 | -1.48 to -0.49 ^**^ | -0.20 | -0.26 to -0.15 ^**^ | -0.26 | -0.32 to -0.20 ^**^ |
| Education → registration in primary care | 0.17 | 0.09 to 0.31 ^**^ | 0.04 | 0.01 to 0.06 ^**^ | 0.06 | 0.04 to 0.09 ^**^ |
| Sex → no use | 0.20 | 0.08 to 0.50 ^**^ | - | - | 0.06 | 0.02 to 0.14 ^**^ |
| Sex → need | - | - | 0.38 | 0.23 to 0.58 ^**^ | 0.36 | 0.24 to 0.52 ^**^ |
| Sex → social network | -0.59 | -0.63 to -0.54 ^**^ | -0.39 | -0.42 to -0.37 ^**^ | -0.43 | -0.45 to -0.41 ^**^ |
| Sex → enabling financing | 1.31 | 0.92 to 2.14 ^**^ | 0.40 | 0.35 to 0.45 ^**^ | 0.50 | 0.45 to 0.56 ^**^ |
| Sex → enabling organisation | 1.36 | 0.90 to 2.20 ^**^ | 0.35 | 0.32 to 0.39 ^**^ | 0.44 | 0.41 to 0.49 ^**^ |
| Sex → registration in primary care | -0.20 | -0.38 to -0.10 ^**^ | -0.08 | -0.10 to -0.07 ^**^ | -0.12 | -0.14 to -0.11 ^**^ |
| Age → no use | 0.41 | 0.28 to 0.69 ^**^ | 0.12 | 0.09 to 0.15 ^**^ | 0.23 | 0.18 to 0.33 ^**^ |
| Age → need | 0.68 | 0.65 to 0.72 ^**^ | 1.05 | 0.92 to 1.25 ^**^ | 1.00 | 0.90 to 1.15 ^**^ |
| Age → social network | -0.46 | -0.52 to -0.38 ^**^ | -0.10 | -0.14 to -0.07 ^**^ | -0.16 | -0.18 to -0.13 ^**^ |
| Age → enabling financing | 1.30 | 0.93 to 2.05 ^**^ | 0.43 | 0.39 to 0.48 ^**^ | 0.49 | 0.45 to 0.54 ^**^ |
| Age → enabling organisation | 1.23 | 0.82 to 2.00 ^**^ | 0.32 | 0.29 to 0.36 ^**^ | 0.39 | 0.35 to 0.42 ^**^ |
| Age → registration in primary care | -0.18 | -0.36 to -0.10 ^**^ | -0.07 | -0.09 to -0.06 ^**^ | -0.10 | -0.12 to -0.09 ^**^ |
| **Indirect effects** |  |  |  |  |  |  |
| Social network → no use | -0.61 | -1.15 to -0.37 ^**^ | -0.27 | -0.30 to -0.23 ^**^ | -0.44 | -0.64 to -0.33 ^**^ |
| Social network → need | -0.17 | -0.34 to -0.09 ^**^ | -1.10 | -1.67 to -0.71 ^**^ | -0.90 | -1.24 to -0.65 ^**^ |
| Enabling financing → no use | 0.02 | 0.01 to 0.03 ^**^ | 0.07 | 0.03 to 0.12 ^**^ | 0.10 | 0.06 to 0.17 ^**^ |
| Enabling organisation → no use | - | - | 0.01 | 0.00 to 0.01 ^**^ | 0.02 | 0.01 to 0.02 ^**^ |
| Registration in primary care → no use | -0.01 | -0.01 to 0.00 ^**^ | 0.00 | -0.01 to 0.00 ^**^ | -0.01 | -0.01 to -0.01 ^**^ |
| Education → no use | 0.05 | -0.03 to 0.24 ^n.s.^ | -0.03 | -0.05 to -0.02 ^**^ | 0.00 | -0.03 to 0.05 ^n.s.^ |
| Education → need | -0.13 | -0.15 to -0.12 ^**^ | 0.06 | -0.03 to 0.20 ^n.s.^ | 0.04 | -0.05 to 0.15 ^n.s.^ |
| Education → enabling financing | 1.04 | 0.74 to 1.68 ^**^ | 0.86 | 0.80 to 0.93 ^**^ | 0.89 | 0.83 to 0.97 ^**^ |
| Education → enabling organisation | 1.08 | 0.75 to 1.76 ^**^ | 0.63 | 0.59 to 0.70 ^**^ | 0.71 | 0.66 to 0.77 ^**^ |
| Education → registration in primary care | -0.19 | -0.32 to -0.12 ^**^ | -0.22 | -0.24 to -0.19 ^**^ | -0.25 | -0.28 to -0.23 ^**^ |
| Sex → no use | -0.27 | -0.58 to -0.15 ^**^ | -0.04 | -0.05 to -0.03 ^**^ | -0.12 | -0.19 to -0.07 ^**^ |
| Sex → need | 0.12 | 0.11 to 0.14 ^**^ | -0.30 | -0.51 to -0.16 ^**^ | -0.28 | -0.44 to -0.16 ^**^ |
| Sex → enabling financing | -1.32 | -2.14 to -0.93 ^**^ | -0.48 | -0.53 to -0.44 ^**^ | -0.56 | -0.61 to -0.51 ^**^ |
| Sex → enabling organisation | -1.38 | -2.23 to -0.92 ^**^ | -0.36 | -0.40 to -0.33 ^**^ | -0.45 | -0.49 to -0.41 ^**^ |
| Sex → registration in primary care | 0.24 | 0.14 to 0.42 ^**^ | 0.12 | 0.11 to 0.14 ^**^ | 0.16 | 0.14 to 0.18 ^**^ |
| Age → no use | -0.45 | -0.74 to -0.32 ^**^ | -0.14 | -0.17 to -0.11 ^**^ | -0.26 | -0.35 to -0.20 ^**^ |
| Age → need | 0.07 | 0.05 to 0.10 ^**^ | -0.37 | -0.57 to -0.23 ^**^ | -0.31 | -0.46 to -0.21 ^**^ |
| Age → enabling financing | -1.03 | -1.78 to -0.67 ^**^ | -0.12 | -0.17 to -0.09 ^**^ | -0.20 | -0.25 to -0.16 ^**^ |
| Age → enabling organisation | -1.07 | -1.82 to -0.66 ^**^ | -0.09 | -0.13 to -0.07 ^**^ | -0.16 | -0.20 to -0.13 ^**^ |
| Age → registration in primary care | 0.19 | 0.11 to 0.36 ^**^ | 0.03 | 0.02 to 0.04 ^**^ | 0.06 | 0.05 to 0.07 ^**^ |
| **Total effects** |  |  |  |  |  |  |
| Need → no use | -0.23 | -0.32 to -0.17 ^**^ | -0.08 | -0.11 to -0.05 ^**^ | -0.16 | -0.22 to -0.13 ^**^ |
| Social network → no use | -0.21 | -0.37 to -0.09 ^**^ | -0.13 | -0.15 to -0.10 ^**^ | -0.15 | -0.17 to -0.12 ^**^ |
| Social network → need | -0.37 | -0.53 to -0.30 ^**^ | -0.14 | -0.19 to -0.09 ^**^ | -0.15 | -0.31 to -0.09 ^**^ |
| Social network → enabling financing | 2.25 | 1.61 to 3.66 ^**^ | 1.24 | 1.17 to 1.31 ^**^ | 1.30 | 0.69 to 1.39 ^**^ |
| Social network → enabling organisation | 2.35 | 1.62 to 3.80 ^**^ | 0.91 | 0.86 to 0.98 ^**^ | 1.04 | 0.45 to 1.11 ^**^ |
| Social network → registration in primary care | -0.41 | -070 to -0.25 ^**^ | -0.31 | -0.34 to -0.28 ^**^ | -0.37 | -0.40 to -0.34 ^**^ |
| Enabling financing → no use | -0.30 | -0.42 to -0.25 ^**^ | -0.17 | -0.21 to -0.12 ^**^ | -0.27 | -0.36 to -0.20 ^**^ |
| Enabling financing → need | -0.07 | -0.10 to -0.04 ^**^ | -0.81 | -1.24 to -0.53 ^**^ | -0.60 | -0.84 to -0.44 ^**^ |
| Enabling organisation → no use | - | - | 0.01 | 0.00 to 0.01 ^**^ | 0.02 | 0.01 to 0.02 ^**^ |
| Enabling organisation → need | - | - | -0.08 | -0.11 to -0.06 ^**^ | -0.09 | -0.12 to -0.07 ^**^ |
| Registration in primary care → no use | -0.05 | -0.07 to -0.03 ^**^ | -0.03 | -0.04 to -0.02 ^**^ | -0.04 | -0.04 to -0.03 ^**^ |
| Registration in primary care → need | 0.02 | 0.01 to 0.04 ^**^ | 0.05 | 0.04 to 0.06 ^**^ | 0.05 | 0.04 to 0.06 ^**^ |
| Education → no use | -0.14 | -0.16 to -0.12 ^**^ | -0.14 | -0.15 to -0.13 ^**^ | -0.16 | -0.17 to -0.15 ^**^ |
| Education → need | -0.13 | -0.15 to -0.12 ^**^ | -0.30 | -0.31 to -0.29 ^**^ | -0.30 | -0.31 to -0.29 ^**^ |
| Education → social network | 0.46 | 0.40 to 0.52 ^**^ | 0.70 | 0.67 to 0.72 ^**^ | 0.68 | 0.66 to 0.70 ^**^ |
| Education → enabling financing | 0.54 | 0.51 to 0.57 ^**^ | 0.68 | 0.67 to 0.70 ^**^ | 0.70 | 0.69 to 0.71 ^**^ |
| Education → enabling organisation | 0.25 | 0.23 to 0.27 ^**^ | 0.44 | 0.43 to 0.44 ^**^ | 0.45 | 0.45 to 0.46 ^**^ |
| Education → registration in primary care | -0.01 | -0.04 to 0.01 ^n.s.^ | -0.18 | -0.19 to -0.17^**^ | -0.19 | -0.19 to 0.18 ^**^ |
| Sex → no use | -0.07 | -0.08 to -0.05 ^**^ | -0.04 | -0.05 to -0.03 ^**^ | -0.05 | -0.06 to -0.04 ^**^ |
| Sex → need | 0.12 | 0.11 to 0.14 ^**^ | 0.07 | 0.07 to 0.08 ^**^ | 0.08 | 0.07 to 0.09 ^**^ |
| Sex → social network | -0.59 | -0.63 to -0.54 ^**^ | -0.39 | -0.42 to -0.37 ^**^ | -0.43 | -0.45 to -0.41 ^**^ |
| Sex → enabling financing | 0.00 | -0.03 to 0.02 ^n.s.^ | -0.09 | -0.10 to -0.07 ^**^ | -0.06 | -0.08 to -0.05 ^**^ |
| Sex → enabling organisation | -0.01 | -0.03 to 0.01 ^n.s.^ | -0.01 | -0.01 to 0.00 ^n.s.^ | 0.00 | -0.01 to 0.00 ^n.s.^ |
| Sex → registration in primary care | 0.04 | 0.02 to 0.06 ^**^ | 0.04 | 0.03 to 0.05 ^**^ | 0.03 | 0.03 to 0.04 ^**^ |
| Age → no use | -0.04 | -0.06 to -0.02 ^**^ | -0.02 | -0.03 to -0.01 ^**^ | -0.03 | -0.04 to -0.02 ^**^ |
| Age → social network | -0.46 | -0.52 to -0.38 ^**^ | -0.10 | -0.14 to -0.07 ^**^ | -0.16 | -0.18 to -0.13 ^**^ |
| Age → need | 0.76 | 0.73 to 0.79 ^**^ | 0.68 | 0.67 to 0.69 ^**^ | 0.69 | 0.68 to 0.70 ^**^ |
| Age → enabling financing | 0.27 | 0.23 to 0.31 ^**^ | 0.31 | 0.30 to 0.32 ^**^ | 0.29 | 0.28 to 0.30 ^**^ |
| Age → enabling organisation | 0.16 | 0.14 to 0.18 ^**^ | 0.23 | 0.22 to 0.24 ^**^ | 0.23 | 0.22 to 0.23 ^**^ |
| Age → registration in primary care | 0.01 | -0.02 to 0.03 ^n.s.^ | -0.04 | -0.05 to -0.03 ^**^ | -0.04 | -0.05 to -0.04 ^**.^ |

β = bootstrapped standardised estimate

^n.s.^ non-significant

^**^ P<0.01
