# Supplementary material for: Utilisation of dental services by Brazilian adults in rural and urban areas: a multi-group structural equation analysis using the Andersen behavioural model
Source: BMC Public Health. 2020 Jun 17;20:953. doi: 10.1186/s12889-020-09100-x (PMC7301519; doi:10.1186/s12889-020-09100-x)
Supplement: Supplementary file 2 — Additional file 2. Direct, indirect and total standardised effects on the structural equation model for the interval since the last dental visit outcome in rural and urban contexts. [file 12889_2020_9100_MOESM2_ESM.docx]

Additional file 2. Direct, indirect and total standardised effects on the structural equation model for the interval since the last dental visit outcome in rural and urban contexts.

| **Parameter** | **Rural** | | **Urban** | | **Total sample** | |
| --- | --- | --- | --- | --- | --- | --- |
|  | **β** | **Bias-corrected**  **95% CI** | **β** | **Bias-corrected**  **95% CI** | **β** | **Bias-corrected**  **95% CI** |
| **Direct effects** |  |  |  |  |  |  |
| Need → interval since last dental visit | 0.41 | 0.39 to 0.43 ^**^ | 0.82 | 0.58 to 1.33 ^**^ | 0.82 | 0.61 to 1.19 ^**^ |
| Social network → interval since last dental visit | - | - | -2.59 | -4.74 to -1.66 ^**^ | -2.53 | -3.65 to -1.86 ^**^ |
| Social network → need | - | - | 0.95 | 0.50 to 1.57 ^**^ | 0.85 | 0.50 to 1.23 ^**^ |
| Social network → enabling financing | 2.40 | 1.64 to 4.16 ^**^ | 1.20 | 1.13 to 1.27 ^**^ | 1.23 | 1.21 to 1.36 ^**^ |
| Social network → enabling organisation | 1.92 | 1.34 to 3.29 ^**^ | 0.88 | 0.83 to 0.94 ^**^ | 1.00 | 0.94 to 1.07 ^**^ |
| Social network → registration in primary care | -0.47 | -0.85 to -0.29 ^**^ | -0.31 | -0.33 to -0.28 ^**^ | -0.36 | -0.39 to -0.34 ^**^ |
| Enabling financing → interval since last dental visit | -0.21 | -0.23 to -0.19 ^**^ | 1.54 | 0.89 to 3.26 ^**^ | 1.25 | 0.81 to 2.00 ^**^ |
| Enabling financing → need | -0.11 | -0.14 to -0.08 ^**^ | -0.82 | -1.32 to -0.53 ^**^ | -0.68 | -0.91 to -0.48 ^**^ |
| Enabling organisation → interval since last dental visit | - | - | 0.16 | 0.11 to 0.23 ^**^ | 0.21 | 0.16 to 0.29 ^**^ |
| Enabling organisation → need | -0.03 | -0.05 to -0.01 ^**^ | -0.09 | -0.12 to -0.06 ^**^ | -0.10 | -0.13 to -0.07 ^**^ |
| Registration in primary care → interval since last dental visit | -0.07 | -0.09 to -0.05 ^**^ | -0.11 | -0.13 to -0.08 ^**^ | -0.12 | -0.15 to -0.10 ^**^ |
| Registration in primary care → need | 0.02 | 0.01 to 0.04 ^**^ | 0.05 | 0.04 to 0.06 ^**^ | 0.05 | 0.04 to 0.06 ^**^ |
| Education → interval since last dental visit | - | - | 0.65 | 0.38 to 1.18 ^**^ | 0.72 | 0.49 to 1.08 ^**^ |
| Education → need | -0.08 | -0.11 to -0.06 ^**^ | -0.36 | -0.51 to -0.27 ^**^ | -0.37 | -0.48 to -0.28 ^**^ |
| Education → social network | 0.48 | 0.41 to 0.54 ^**^ | 0.70 | 0.68 to 0.72 ^**^ | 0.69 | 0.67 to 0.71 ^**^ |
| Education → enabling financing | -0.61 | -1.54 to -0.22 ^**^ | -0.17 | -0.23 to -0.09 ^**^ | -0.19 | -0.26 to -0.12 ^**^ |
| Education → enabling organisation | -0.66 | -1.28 to -0.36 ^**^ | -0.18 | -0.24 to -0.13 ^**^ | -0.24 | -0.31 to -0.19 ^**^ |
| Education → registration in primary care | 0.20 | 0.11 to 0.38 ^**^ | 0.03 | 0.01 to 0.05 ^*^ | 0.06 | 0.03 to 0.09 ^**^ |
| Sex → interval since last dental visit | -0.12 | -0.14 to -0.10 ^**^ | -1.00 | -1.75 to -0.67 ^**^ | -1.13 | -1.61 to -0.85 ^**^ |
| Sex → need | 0.12 | 0.10 to 0.13 ^**^ | 0.37 | 0.22 to 0.59 ^**^ | 0.40 | 0.26 to 0.56 ^**^ |
| Sex → social network | -0.59 | -0.63 to -0.54 ^**^ | -0.39 | -0.41 to -0.37 ^**^ | -0.43 | -0.45 to -0.41 ^**^ |
| Sex → enabling financing | 1.40 | 0.92 to 2.45 ^**^ | 0.38 | 0.33 to 0.43 ^**^ | 0.48 | 0.43 to 0.54 ^**^ |
| Sex → enabling organisation | 1.11 | 0.75 to 1.90 ^**^ | 0.34 | 0.30 to 0.37 ^**^ | 0.42 | 0.39 to 0.47 ^**^ |
| Sex → registration in primary care | -0.24 | -0.47 to -0.13 ^**^ | -0.08 | -0.10 to -0.07 ^**^ | -0.12 | -0.14 to -0.10 ^**^ |
| Age → interval since last dental visit | - | - | -1.05 | -2.05 to -0.61 ^**^ | -1.11 | -1.71 to -0.75 ^**^ |
| Age → need | 0.81 | 0.79 to 0.84 ^**^ | 1.00 | 0.88 to 1.20 ^**^ | 1.01 | 0.90 to 1.13 ^**^ |
| Age → social network | -0.43 | -0.51 to -0.36 ^**^ | -0.06 | -0.09 to -0.02 ^**^ | -0.12 | -0.16 to -0.09 ^**^ |
| Age → enabling financing | 1.32 | 0.90 to 2.27 ^**^ | 0.37 | 0.33 to 0.42 ^**^ | 0.45 | 0.41 to 0.50 ^**^ |
| Age → enabling organisation | 0.99 | 0.69 to 1.72 ^**^ | 0.28 | 0.25 to 0.31 ^**^ | 0.35 | 0.32 to 0.40 ^**^ |
| Age → registration in primary care | -0.20 | -0.41 to -0.11 ^**^ | -0.06 | -0.08 to -0.05 ^**^ | -0.09 | -0.11 to -0.08 ^**^ |
| **Indirect effects** |  |  |  |  |  |  |
| Social network → interval since last dental visit | -0.62 | -1.07 to -0.42 ^**^ | 1.92 | 1.06 to 4.06 ^**^ | 1.75 | 1.12 to 2.81 ^**^ |
| Social network → need | -0.34 | -0.56 to -0.22 ^**^ | -1.07 | -1.74 to -0.70 ^**^ | -0.99 | -1.35 to -0.69 ^**^ |
| Enabling financing → interval since last dental visit | -0.05 | -0.06 to -0.03 ^**^ | -0.67 | -1.78 to -0.32 ^**^ | -0.55 | -1.04 to -0.29 ^**^ |
| Enabling organisation → interval since last dental visit | -0.01 | -0.02 to 0.00 ^**^ | -0.07 | -0.14 to -0.04 ^**^ | -0.08 | -0.15 to -0.05 ^**^ |
| Registration in primary care → interval since last dental visit | 0.01 | 0.00 to 0.02 ^**^ | 0.04 | 0.02 to 0.07 ^**^ | 0.04 | 0.03 to 0.07 ^**^ |
| Education → interval since last dental visit | -0.17 | -0.19 to -0.16 ^**^ | -0.93 | -1.45 to -0.66 ^**^ | -1.01 | -1.37 to -0.79 ^**^ |
| Education → need | -0.07 | -0.08 to -0.05 ^**^ | 0.06 | -0.04 to 0.21 ^n.s.^ | 0.07 | -0.03 to 0.17 ^n.s.^ |
| Education → enabling financing | 1.14 | 0.76 to 2.06 ^**^ | 0.84 | 0.77 to 0.91 ^**^ | 0.88 | 0.82 to 0.95 ^**^ |
| Education → enabling organisation | 0.91 | 0.62 to 1.54 ^**^ | 0.62 | 0.57 to 0.67 ^**^ | 0.69 | 0.64 to 0.75 ^**^ |
| Education → registration in primary care | -0.23 | -0.40 to -0.13 ^**^ | -0.21 | -0.24 to -0.19 ^**^ | -0.25 | -0.28 to -0.23 ^**^ |
| Sex → interval since last dental visit | 0.05 | 0.04 to 0.06 ^**^ | 0.93 | 0.60 to 1.68 ^**^ | 1.06 | 0.78 to 1.54 ^**^ |
| Sex → need | 0.00 | 0.00 to 0.01 ^n.s.^ | -0.29 | -0.52 to -0.15 ^**^ | -0.32 | -0.47 to -0.18 ^**^ |
| Sex → enabling financing | -1.41 | -2.49 to -0.94 ^**^ | -0.47 | -0.52 to -0.42 ^**^ | -0.55 | -0.61 to -0.50 ^**^ |
| Sex → enabling organisation | -1.13 | -1.90 to -0.76 ^**^ | -0.34 | -0.38 to -0.31 ^**^ | -0.43 | -0.47 to -0.39 ^**^ |
| Sex → registration in primary care | 0.28 | 0.17 to 0.52 ^**^ | 0.12 | 0.11 to 0.13 ^**^ | 0.16 | 0.14 to 0.17 ^**^ |
| Age → interval since last dental visit | 0.26 | 0.24 to 0.28 ^**^ | 1.22 | 0.78 to 2.23 ^**^ | 1.29 | 0.92 to 1.88 ^**^ |
| Age → need | -0.04 | -0.05 to -0.03 ^**^ | -0.33 | -0.53 to -0.20 ^**^ | -0.33 | -0.45 to -0.22 ^**^ |
| Age → enabling financing | -1.04 | -2.03 to -0.63 ^**^ | -0.07 | -0.11 to -0.03 ^**^ | -0.16 | -0.21 to -0.11 ^**^ |
| Age → enabling organisation | -0.83 | -1.57 to -0.53 ^**^ | -0.05 | -0.08 to -0.02 ^**^ | -0.12 | -0.16 to -0.09 ^**^ |
| Age → registration in primary care | 0.20 | 0.12 to 0.42 ^**^ | 0.02 | 0.01 to 0.03 ^**^ | 0.05 | 0.03 to 0.06 ^**^ |
| **Total effects** |  |  |  |  |  |  |
| Need → interval since last dental visit | 0.41 | 0.39 to 0.43 ^**^ | 0.82 | 0.58 to 1.33 ^**^ | 0.82 | 0.61 to 1.19 ^**^ |
| Social network → interval since last dental visit | -0.62 | -1.07 to -0.42 ^**^ | -0.68 | -0.76 to -0.60 ^**^ | -0.78 | -0.90 to -0.69 ^**^ |
| Social network → need | -0.34 | -0.56 to -0.22 ^**^ | -0.13 | -0.18 to -0.07 ^**^ | -0.14 | -0.19 to -0.07 ^**^ |
| Social network → enabling financing | 2.40 | 1.64 to 4.16 ^**^ | 1.20 | 1.13 to 1.27 ^**^ | 1.23 | 1.21 to 1.36 ^**^ |
| Social network → enabling organisation | 1.92 | 1.34 to 3.29 ^**^ | 0.88 | 0.83 to 0.94 ^**^ | 1.00 | 0.94 to 1.07 ^**^ |
| Social network → registration in primary care | -0.47 | -0.85 to -0.29 ^**^ | -0.31 | -0.33 to -0.28 ^**^ | -0.36 | -0.39 to -0.33 ^**^ |
| Enabling financing → interval since last dental visit | -0.26 | -0.28 to -0.24 ^**^ | 0.87 | 0.51 to 1.65 ^**^ | 0.70 | 0.44 to 1.05 ^**^ |
| Enabling financing → need | -0.11 | -0.14 to -0.08 ^**^ | -0.82 | -1.32 to -0.53 ^**^ | -0.68 | -0.91 to -0.48 ^**^ |
| Enabling organisation → interval since last dental visit | -0.01 | -0.02 to 0.00 ^**^ | 0.09 | 0.06 to 0.13 ^**^ | 0.13 | 0.09 to 0.18 ^**^ |
| Enabling organisation → need | -0.03 | -0.05 to -0.01 ^**^ | -0.09 | -0.12 to -0.06 ^**^ | -0.10 | -0.13 to -0.07 ^**^ |
| Registration in primary care → interval since last dental visit | -0.06 | -0.07 to -0.04 ^**^ | -0.07 | -0.08 to -0.06 ^**^ | -0.08 | -0.09 to -0.07 ^**^ |
| Registration in primary care → need | 0.02 | 0.01 to 0.04 ^**^ | 0.05 | 0.04 to 0.06 ^**^ | 0.05 | 0.04 to 0.06 ^**^ |
| Education → interval since last dental visit | -0.17 | -0.19 to -0.16 ^**^ | -0.29 | -0.30 to -0.28 ^**^ | -0.30 | -0.30 to -0.29 ^**^ |
| Education → need | -0.15 | -0.17 to -0.13 ^**^ | -0.30 | -0.31 to -0.29 ^**^ | -0.31 | -0.32 to -0.30 ^**^ |
| Education → social network | 0.48 | 0.41 to 0.54 ^**^ | 0.70 | 0.68 to 0.72 ^**^ | 0.69 | 0.67 to 0.71 ^**^ |
| Education → enabling financing | 0.54 | 0.51 to 0.56 ^**^ | 0.67 | 0.66 to 0.69 ^**^ | 0.69 | 0.68 to 0.70 ^**^ |
| Education → enabling organisation | 0.25 | 0.23 to 0.28 ^**^ | 0.43 | 0.43 to 0.44 ^**^ | 0.45 | 0.44 to 0.46 ^**^ |
| Education → registration in primary care | -0.02 | -0.04 to 0.00 ^**^ | -0.19 | -0.19 to -0.18 ^**^ | -0.19 | -0.20 to -0.18 ^**^ |
| Sex → interval since last dental visit | -0.07 | -0.09 to -0.05 ^**^ | -0.07 | -0.08 to -0.06 ^**^ | -0.07 | -0.08 to -0.06 ^**^ |
| Sex → need | 0.12 | 0.11 to 0.14 ^**^ | 0.07 | 0.07 to 0.08 ^**^ | 0.08 | 0.07 to 0.09 ^**^ |
| Sex → social network | -0.59 | -0.63 to -0.54 ^**^ | -0.39 | -0.41 to -0.37 ^**^ | -0.43 | -0.45 to -0.41 ^**^ |
| Sex → enabling financing | -0.02 | -0.04 to 0.01 ^n.s.^ | -0.09 | -0.10 to -0.08 ^**^ | -0.07 | -0.08 to -0.05 ^**^ |
| Sex → enabling organisation | -0.02 | -0.03 to 0.00 ^n.s.^ | -0.01 | -0.02 to 0.00 ^n.s.^ | -0.01 | -0.01 to 0.00 ^n.s.^ |
| Sex → registration in primary care | 0.04 | 0.02 to 0.06 ^**^ | 0.04 | 0.03 to 0.05 ^**^ | 0.04 | 0.03 to 0.04 ^**^ |
| Age → interval since last dental visit | 0.26 | 0.24 to 0.28 ^**^ | 0.17 | 0.16 to 0.18 ^**^ | 0.18 | 0.17 to 0.19 ^**^ |
| Age → social network | -0.43 | -0.51 to -0.36 ^**^ | -0.06 | -0.09 to -0.02 ^**^ | -0.12 | -0.16 to -0.09 ^**^ |
| Age → need | 0.78 | 0.76 to 0.80 ^**^ | 0.68 | 0.67 to 0.69 ^**^ | 0.68 | 0.67 to 0.69 ^**^ |
| Age → enabling financing | 0.27 | 0.23 to 0.31 ^**^ | 0.31 | 0.29 to 0.24 ^**^ | 0.29 | 0.28 to 0.30 ^**^ |
| Age → enabling organisation | 0.16 | 0.14 to 0.18 ^**^ | 0.23 | 0.22 to 0.24 ^**^ | 0.23 | 0.22 to 0.23 ^**^ |
| Age → registration in primary care | 0.00 | -0.02 to 0.02 ^n.s.^ | -0.04 | -0.05 to -0.04 ^**^ | -0.05 | -0.06 to -0.04 ^**^ |

β = bootstrapped standardised estimate

^n.s.^ non-significant

^*^ P<0.05

^**^ P<0.01
