# Supplementary material for: Utilisation of dental services by Brazilian adults in rural and urban areas: a multi-group structural equation analysis using the Andersen behavioural model
Source: BMC Public Health. 2020 Jun 17;20:953. doi: 10.1186/s12889-020-09100-x (PMC7301519; doi:10.1186/s12889-020-09100-x)
Supplement: Supplementary file 3 — Additional file 3. Calculation of specific indirect paths of total indirect effects for the non-utilisation of dental services. [file 12889_2020_9100_MOESM3_ESM.docx]

Additional file 3. Calculation of specific indirect paths of total indirect effects for the non-utilisation of dental services.

| **Paths** | **β** | |
| --- | --- | --- |
|  | **Rural** | **Urban** |
| Social network → need → no use | 0.048 | -0.078 |
| Social network → registration in primary care → no use | 0.016 | 0.009 |
| Social network → Enabling financing → no use | -0.720 | -0.285 |
| Social network → registration in primary care → need → no use | 0.002 | 0.001 |
| Social network → Enabling financing → need → no use | 0.036 | 0.080 |
| Social network → Enabling organisation → need → no use | - | 0.006 |
| Social network → registration in primary care → need | -0.008 | -0.016 |
| Social network → Enabling financing → need | -0.158 | -1.004 |
| Social network → Enabling organisation → need | - | -0.073 |
| Enabling financing → need → no use | 0.016 | 0.065 |
| Enabling organisation → need → no use | - | 0.006 |
| Registration in primary care → need → no use | -0.005 | -0.004 |
| Education → need → no use | - | 0.029 |
| Education → enabling financing → no use | 0.157 | 0.041 |
| Education → enabling financing → need → no use | -0.008 | -0.012 |
| Education → enabling organisation → need → no use | - | -0.001 |
| Education → social network → no use | 0.189 | 0.097 |
| Education → social network → enabling financing → no use | -0.331 | -0.197 |
| Education → social network → enabling financing → need → no use | 0.017 | 0.055 |
| Education → social network → enabling organisation → need → no use | - | 0.004 |
| Education → social network → need → no use | 0.022 | -0.052 |
| Education → social network → registration in primary care → no use | 0.008 | 0.006 |
| Education → social network → registration in primary care → need → no use | 0.001 | 0.001 |
| Education → registration in primary care → no use | -0.007 | -0.001 |
| Education → registration in primary care → need → no use | -0.001 | 0.000 |
| Education → enabling financing → need | 0.034 | 0.146 |
| Education → enabling organisation → need | - | 0.016 |
| Education → social network → need | -0.097 | 0.656 |
| Education → social network → enabling financing → need | -0.072 | -0.693 |
| Education → social network → enabling organisation → need | - | -0.050 |
| Education → social network → registration in primary care → need | -0.004 | -0.011 |
| Education → registration in primary care → need | 0.003 | 0.002 |
| Education → social network → enabling financing | 1.035 | 0.856 |
| Education → social network → enabling organisation | 1.081 | 0.628 |
| Education → social network → registration in primary care | -0.189 | -0.214 |
| Sex → need → no use | - | -0.030 |
| Sex → enabling financing → no use | -0.419 | -0.092 |
| Sex → enabling financing → need → no use | 0.021 | 0.026 |
| Sex → enabling organisation → need → no use | - | 0.002 |
| Sex → social network → no use | -0.242 | -0.055 |
| Sex → social network → enabling financing → no use | 0.425 | 0.111 |
| Sex → social network → enabling financing → need → no use | -0.021 | -0.031 |
| Sex → social network → enabling organisation → need → no use | - | -0.002 |
| Sex → social network → need → no use | -0.028 | 0.030 |
| Sex → social network → registration in primary care → no use | -0.010 | -0.004 |
| Sex → social network → registration in primary care → need → no use | -0.001 | 0.000 |
| Sex → registration in primary care → no use | 0.008 | 0.002 |
| Sex → registration in primary care → need → no use | 0.001 | 0.000 |
| Sex → enabling financing → need | -0.092 | -0.324 |
| Sex → enabling organisation → need | - | -0.028 |
| Sex → social network → need | -0.242 | -0.055 |
| Sex → social network → enabling financing → need | 0.093 | 0.392 |
| Sex → social network → enabling organisation → need | - | 0.028 |
| Sex → social network → registration in primary care → need | 0.005 | 0.006 |
| Sex → registration in primary care → need | -0.004 | -0.004 |
| Sex → social network → enabling financing | -1.328 | -0.484 |
| Sex → social network → enabling organisation | -1.387 | -0.355 |
| Sex → social network → registration in primary care | 0.242 | 0.121 |
| Age → need → no use | -0.156 | -0.084 |
| Age → enabling financing → no use | -0.416 | -0.099 |
| Age → enabling financing → need → no use | 0.021 | 0.028 |
| Age → enabling organisation → need → no use | - | 0.002 |
| Age → social network → no use | -0.189 | -0.014 |
| Age → social network → enabling financing → no use | 0.331 | 0.029 |
| Age → social network → enabling financing → need → no use | -0.017 | -0.008 |
| Age → social network → enabling organisation → need → no use | - | -0.001 |
| Age → social network → need → no use | -0.022 | 0.008 |
| Age → social network → registration in primary care → no use | -0.008 | -0.001 |
| Age → social network → registration in primary care → need → no use | -0.001 | 0.000 |
| Age → registration in primary care → no use | 0.007 | 0.002 |
| Age → registration in primary care → need → no use | 0.001 | 0.000 |
| Age → enabling financing → need | -0.091 | -0.348 |
| Age → enabling organisation → need | - | -0.026 |
| Age → social network → need | 0.097 | -0.095 |
| Age → social network → enabling financing → need | 0.072 | 0.100 |
| Age → social network → enabling organisation → need | - | 0.007 |
| Age → social network → registration in primary care → need | 0.004 | 0.002 |
| Age → registration in primary care → need | -0.004 | -0.004 |
| Age → social network → enabling financing | -1.035 | -0.124 |
| Age → social network → enabling organisation | -1.081 | -0.091 |
| Age → social network → registration in primary care | 0.187 | 0.031 |

β = bootstrapped standardised estimate
