# Supplementary material for: Utilisation of dental services by Brazilian adults in rural and urban areas: a multi-group structural equation analysis using the Andersen behavioural model
Source: BMC Public Health. 2020 Jun 17;20:953. doi: 10.1186/s12889-020-09100-x (PMC7301519; doi:10.1186/s12889-020-09100-x)
Supplement: Supplementary file 4 — Additional file 4. Calculation of specific indirect paths of total indirect effects for the interval since the last dental visit. [file 12889_2020_9100_MOESM4_ESM.docx]

Additional file 4. Calculation of specific indirect paths of total indirect effects for the interval since the last dental visit.

| **Paths** | **β** | |
| --- | --- | --- |
|  | **Rural** | **Urban** |
| Social network → need → interval since last dental visit | - | 0.779 |
| Social network → registration in primary care → interval since last dental visit | 0.033 | 0.034 |
| Social network → Enabling financing → interval since last dental visit | -0.504 | 1.848 |
| Social network → Enabling organisation → interval since last dental visit | - | -0.141 |
| Social network → Enabling financing → need → interval since last dental visit | -0.108 | -0.807 |
| Social network → Enabling organisation → need → interval since last dental visit | -0.024 | -0.065 |
| Social network → registration in primary care → need → interval since last dental visit | -0.004 | -0.013 |
| Social network → registration in primary care → need | -0.009 | -0.016 |
| Social network → Enabling financing → need | -0.264 | -0.984 |
| Social network → Enabling organisation → need | -0.058 | -0.079 |
| Enabling financing → need → interval since last dental visit | -0.045 | -0.672 |
| Enabling organisation → need → interval since last dental visit | -0.012 | -0.074 |
| Registration in primary care → need → interval since last dental visit | 0.008 | 0.041 |
| Education → need → interval since last dental visit | -0.033 | -0.295 |
| Education → Enabling financing → interval since last dental visit | 0.128 | -0.262 |
| Education → Enabling financing → need → interval since last dental visit | 0.028 | 0.114 |
| Education → Enabling organisation → interval since last dental visit | - | -0.029 |
| Education → Enabling organisation → need → interval since last dental visit | 0.008 | 0.013 |
| Education → Social network → interval since last dental visit | - | -1.813 |
| Education → Social network → Enabling financing → interval since last dental visit | -0.242 | 1.294 |
| Education → Social network → Enabling financing → need → interval since last dental visit | -0.052 | -0.565 |
| Education → Social network → Enabling organisation → interval since last dental visit | - | 0.099 |
| Education → Social network → Enabling organisation → need → interval since last dental visit | -0.011 | -0.045 |
| Education → Social network → need → interval since last dental visit | - | 0.545 |
| Education → Social network → registration in primary care → interval since last dental visit | 0.016 | 0.024 |
| Education → Social network → registration in primary care → need → interval since last dental visit | -0.002 | -0.009 |
| Education → registration in primary care → interval since last dental visit | -0.014 | -0.003 |
| Education → registration in primary care → need → interval since last dental visit | 0.002 | 0.001 |
| Education → Enabling financing → need | 0.067 | 0.139 |
| Education → Enabling organisation → need | 0.020 | 0.016 |
| Education → registration in primary care → need | 0.004 | 0.002 |
| Education → Social network → need | - | 0.665 |
| Education → Social network → registration in primary care → need | -0.005 | -0.011 |
| Education → Social network → Enabling financing → need | -0.127 | -0.689 |
| Education → Social network → Enabling organisation → need | -0.028 | -0.055 |
| Education → Social network → Enabling financing | 1.152 | 0.840 |
| Education → Social network → Enabling organisation | 0.922 | 0.616 |
| Education → Social network → registration in primary care | -0.226 | -0.217 |
| Sex → enabling financing → interval since last dental visit | -0.294 | 0.585 |
| Sex → enabling financing → need → interval since last dental visit | -0.063 | -0.256 |
| Sex → enabling organisation → interval since last dental visit | - | 0.054 |
| Sex → enabling organisation → need → interval since last dental visit | -0.014 | -0.025 |
| Sex → need → interval since last dental visit | 0.049 | 0.303 |
| Sex → social network → interval since last dental visit | - | 1.010 |
| Sex → social network → need → interval since last dental visit | - | -0.304 |
| Sex → social network → enabling financing → interval since last dental visit | 0.297 | -0.721 |
| Sex → social network → enabling financing → need → interval since last dental visit | 0.064 | 0.315 |
| Sex → social network → enabling organisation → interval since last dental visit | - | -0.055 |
| Sex → social network → enabling organisation → need → interval since last dental visit | 0.014 | 0.025 |
| Sex → social network → registration in primary care → interval since last dental visit | -0.019 | -0.013 |
| Sex → social network → registration in primary care → need → interval since last dental visit | 0.002 | 0.005 |
| Sex → registration in primary care → interval since last dental visit | 0.017 | 0.009 |
| Sex → registration in primary care → need → interval since last dental visit | -0.002 | -0.003 |
| Sex → enabling financing → need | -0.154 | -0.312 |
| Sex → enabling organisation → need | -0.033 | -0.031 |
| Sex → registration in primary care → need | -0.005 | -0.004 |
| Sex → social network → need | - | 1.010 |
| Sex → social network → enabling financing → need | 0.156 | 0.384 |
| Sex → social network → enabling organisation → need | 0.034 | 0.031 |
| Sex → social network → registration in primary care → need | 0.006 | 0.006 |
| Sex → social network → enabling financing | -1.416 | -0.468 |
| Sex → social network → enabling organisation | -1.133 | -0.343 |
| Sex → social network → registration in primary care | 0.277 | 0.121 |
| Age → enabling financing → interval since last dental visit | -0.277 | 0.057 |
| Age → enabling financing → need → interval since last dental visit | -0.060 | -0.249 |
| Age → enabling organisation → interval since last dental visit | - | 0.045 |
| Age → enabling organisation → need → interval since last dental visit | -0.012 | -0.021 |
| Age → need → interval since last dental visit | 0.332 | 0.820 |
| Age → social network → interval since last dental visit | - | 0.155 |
| Age → social network → need → interval since last dental visit | - | -0.047 |
| Age → social network → enabling financing → interval since last dental visit | 0.216 | -0.111 |
| Age → social network → enabling financing → need → interval since last dental visit | 0.047 | 0.048 |
| Age → social network → enabling organisation → interval since last dental visit | - | -0.008 |
| Age → social network → enabling organisation → need → interval since last dental visit | 0.010 | 0.004 |
| Age → social network → registration in primary care → interval since last dental visit | -0.014 | -0.002 |
| Age → social network → registration in primary care → need → interval since last dental visit | 0.002 | 0.001 |
| Age → registration in primary care → interval since last dental visit | 0.014 | 0.007 |
| Age → registration in primary care → need → interval since last dental visit | -0.002 | -0.002 |
| Age → enabling financing → need | -0.145 | -0.303 |
| Age → enabling organisation → need | -0.030 | -0.025 |
| Age → registration in primary care → need | -0.004 | -0.003 |
| Age → social network → need | - | -0.057 |
| Age → social network → enabling financing → need | 0.114 | 0.059 |
| Age → social network → enabling organisation → need | 0.025 | 0.005 |
| Age → social network → registration in primary care → need | 0.004 | 0.001 |
| Age → social network → enabling financing | -1.032 | -0.072 |
| Age → social network → enabling organisation | -0.826 | -0.053 |
| Age → social network → registration in primary care | 0.202 | 0.019 |

β = bootstrapped standardised estimate
