# Supplementary material for: Utilisation of dental services by Brazilian adults in rural and urban areas: a multi-group structural equation analysis using the Andersen behavioural model
Source: BMC Public Health. 2020 Jun 17;20:953. doi: 10.1186/s12889-020-09100-x (PMC7301519; doi:10.1186/s12889-020-09100-x)
Supplement: Supplementary file 5 — Additional file 5. Direct, indirect and total non-standardised effects on the structural equation model for the non-utilisation of dental services in rural and urban contexts. [file 12889_2020_9100_MOESM5_ESM.docx]

Additional file 5. Direct, indirect and total non-standardised effects on the structural equation model for the non-utilisation of dental services in rural and urban contexts.

| **Parameter** | **Rural** | | **Urban** | | **Total sample** | |
| --- | --- | --- | --- | --- | --- | --- |
|  | **β** | **Bias-corrected**  **95% CI** | **β** | **Bias-corrected**  **95% CI** | **β** | **Bias-corrected**  **95% CI** |
| **Direct effects** |  |  |  |  |  |  |
| Need → no use | -0.30 | -0.42 to -0.22 ^**^ | -0.08 | -0.11 to -0.05 ^**^ | -0.17 | -0.23 to -0.13 ^**^ |
| Social network → no use | 0.99 | 0.30 to 16.57 ^**^ | 0.04 | 0.03 to 0.05 ^**^ | 0.12 | 0.08 to 0.21 ^**^ |
| Social network → need | -0.38 | -8.97 to -0.18 ^**^ | 0.29 | 0.16 to 0.47 ^**^ | 0.30 | 0.18 to 0.46 ^**^ |
| Social network → enabling financing | 79.66 | 30.83 to 1360.93 ^**^ | 13.94 | 12.44 to 15.66 ^**^ | 18.45 | 16.36 to 21.13 ^**^ |
| Social network → enabling organisation | 5.33 | 2.09 to 96.01 ^**^ | 0.79 | 0.70 to 0.89 ^**^ | 1.04 | 0.92 to 1.20 ^**^ |
| Social network → registration in primary care | -1.72 | -29.94 to -0.59 ^**^ | -0.30 | -0.34 to -0.26 ^**^ | -0.43 | -0.49 to -0.37 ^**^ |
| Enabling financing → no use | -0.02 | -0.03 to -0.02 ^**^ | -0.01 | -0.01 to -0.01 ^**^ | -0.01 | -0.01 to -0.01 ^**^ |
| Enabling financing → need | 0.00 | -0.01 to 0.00 ^**^ | -0.02 | -0.03 to -0.01 ^**^ | -0.02 | -0.02 to -0.01 ^**^ |
| Enabling organisation → need | - | - | -0.03 | -0.04 to -0.02 ^**^ | -0.04 | -0.05 to -0.03 ^**^ |
| Registration in primary care → no use | -0.02 | -0.04 to -0.02 ^**^ | -0.01 | -0.01 to -0.01 ^**^ | -0.01 | -0.01 to -0.01 ^**^ |
| Registration in primary care → need | 0.01 | 0.00 to 0.02 ^**^ | 0.02 | 0.01 to 0.02 ^**^ | 0.02 | 0.01 to 0.02 ^**^ |
| Education → no use | -0.01 | -0.02 to -0.01 ^**^ | 0.00 | 0.00 to 0.00 ^**^ | -0.01 | -0.01 to 0.00 ^**^ |
| Education → need | - | - | -0.01 | -0.02 to -0.01 ^**^ | -0.01 | -0.02 to -0.01 ^**^ |
| Education → social network | 0.01 | 0.00 to 0.02 ^**^ | 0.08 | 0.07 to 0.08 ^**^ | 0.06 | 0.05 to 0.07 ^**^ |
| Education → enabling financing | -0.43 | -0.97 to -0.17 ^**^ | -0.22 | -0.30 to -0.14 ^**^ | -0.24 | -0.33 to -0.16 ^**^ |
| Education → enabling organisation | -0.05 | -0.08 to -0.03 ^**^ | -0.02 | -0.02 to -0.01 ^**^ | -0.02 | -0.03 to -0.02 ^**^ |
| Education → registration in primary care | 0.02 | 0.01 to 0.03 ^**^ | 0.00 | 0.00 to 0.01 ^**^ | 0.01 | 0.00 to 0.01 ^**^ |
| Sex → no use | 0.11 | 0.04 to 0.28 ^**^ | - | - | 0.02 | 0.01 to 0.05 ^**^ |
| Sex → need | - | - | 0.12 | 0.08 to 0.19 ^**^ | 0.12 | 0.08 to 0.18 ^**^ |
| Sex → social network | -0.13 | -0.27 to -0.01 ^**^ | -0.41 | -0.45 to -0.37 ^**^ | -0.37 | -0.41 to -0.33 ^**^ |
| Sex → enabling financing | 10.39 | 7.33 to 16.88 ^**^ | 4.73 | 4.20 to 5.37 ^**^ | 6.10 | 5.54 to 6.82 ^**^ |
| Sex → enabling organisation | 0.69 | 0.45 to 1.13 ^**^ | 0.32 | 0.29 to 0.36 ^**^ | 0.38 | 0.35 to 0.42 ^**^ |
| Sex → registration in primary care | -0.19 | -0.36 to -0.10 ^**^ | -0.09 | -0.10 to -0.07 ^**^ | -0.12 | -0.14 to -0.11 ^**^ |
| Age → no use | 0.01 | 0.00 to 0.01 ^**^ | 0.00 | 0.00 to 0.00 ^**^ | 0.00 | 0.00 to 0.00 ^**^ |
| Age → need | 0.01 | 0.01 to 0.01 ^**^ | 0.01 | 0.01 to 0.01 ^**^ | 0.01 | 0.01 to 0.01 ^**^ |
| Age → social network | 0.00 | -0.01 to 0.00 ^**^ | 0.00 | 0.00 to 0.00 ^**^ | 0.00 | 0.00 to 0.00 ^**^ |
| Age → enabling financing | 0.31 | 0.22 to 0.48 ^**^ | 0.15 | 0.14 to 0.17 ^**^ | 0.18 | 0.16 to 0.19 ^**^ |
| Age → enabling organisation | 0.02 | 0.01 to 0.03 ^**^ | 0.01 | 0.01 to 0.01 ^**^ | 0.01 | 0.01 to 0.01 ^**^ |
| Age → registration in primary care | -0.01 | -0.01 to 0.00 ^**^ | 0.00 | 0.00 to 0.00 ^**^ | 0.00 | 0.00 to 0.00 ^**^ |
| **Indirect effects** |  |  |  |  |  |  |
| Social network → no use | -1.49 | -22.88 to -0.52 ^**^ | -0.08 | -0.09 to -0.07 ^**^ | -0.19 | -0.27 to -0.14 ^**^ |
| Social network → need | -0.31 | -4.81 to -0.11 ^**^ | -0.33 | -0.51 to -0.22 ^**^ | -0.36 | -0.51 to -0.25 ^**^ |
| Enabling financing → no use | 0.00 | 0.00 to 0.00 ^**^ | 0.00 | 0.00 to 0.00 ^**^ | 0.00 | 0.00 to 0.01 ^**^ |
| Enabling organisation → no use | - | - | 0.00 | 0.00 to 0.00 ^**^ | 0.01 | 0.00 to 0.01 ^**^ |
| Registration in primary care → no use | 0.00 | -0.01 to 0.00 ^**^ | 0.00 | 0.00 to 0.00 ^**^ | 0.00 | 0.00 to 0.00 ^**^ |
| Education → no use | 0.00 | 0.00 to 0.01 ^n.s.^ | 0.00 | 0.00 to 0.00 ^**^ | 0.00 | 0.00 to 0.00 ^n.s.^ |
| Education → need | -0.01 | -0.01 to -0.01 ^**^ | 0.00 | 0.00 to 0.01 ^n.s.^ | 0.00 | 0.00 to 0.01 ^n.s.^ |
| Education → enabling financing | 0.90 | 0.63 to 1.45 ^**^ | 1.06 | 0.98 to 1.15 ^**^ | 1.10 | 1.02 to 1.19 ^**^ |
| Education → enabling organisation | 0.06 | 0.04 to 0.10 ^**^ | 0.06 | 0.06 to 0.07 ^**^ | 0.06 | 0.06 to 0.07 ^**^ |
| Education → registration in primary care | -0.02 | -0.03 to -0.01 ^**^ | -0.02 | -0.03 to -0.02 ^**^ | -0.03 | -0.03 to -0.02 ^**^ |
| Sex → no use | -0.14 | -0.31 to -0.08 ^**^ | -0.01 | -0.02 to -0.01 ^**^ | -0.04 | -0.07 to -0.03 ^**^ |
| Sex → need | 0.05 | 0.04 to 0.06 ^**^ | -0.10 | -0.16 to -0.05 ^**^ | -0.10 | -0.15 to -0.06 ^**^ |
| Sex → enabling financing | -10.43 | -17.01 to -7.35 ^**^ | -5.76 | -6.33 to -5.21 ^**^ | -6.86 | -7.53 to -6.27 ^**^ |
| Sex → enabling organisation | -0.70 | -1.13 to -0.47 ^**^ | -0.32 | -0.36 to -0.29 ^**^ | -0.39 | -0.43 to -0.35 ^**^ |
| Sex → registration in primary care | 0.23 | 0.13 to 0.39 ^**^ | 0.12 | 0.11 to 0.14 ^**^ | 0.16 | 0.14 to 0.18 ^**^ |
| Age → no use | -0.01 | -0.01 to -0.01 ^**^ | 0.00 | 0.00 to 0.00 ^**^ | 0.00 | 0.00 to 0.00 ^**^ |
| Age → need | 0.00 | 0.00 to 0.00 ^**^ | 0.00 | -0.01 to 0.00 ^**^ | 0.00 | 0.00 to 0.00 ^**^ |
| Age → enabling financing | -0.24 | -0.41 to -0.16 ^**^ | -0.04 | -0.06 to -0.03 ^**^ | -0.07 | -0.09 to -0.06 ^**^ |
| Age → enabling organisation | -0.02 | -0.03 to -0.01 ^**^ | 0.00 | 0.00 to 0.00 ^**^ | 0.00 | -0.01 to 0.00 ^**^ |
| Age → registration in primary care | 0.01 | 0.00 to 0.01 ^**^ | 0.00 | 0.00 to 0.00 ^**^ | 0.00 | 0.00 to 0.00 ^**^ |
| **Total effects** |  |  |  |  |  |  |
| Need → no use | -0.30 | -0.42 to -0.22 ^**^ | -0.08 | -0.11 to -0.05 ^**^ | -0.17 | -0.23 to -0.13 ^**^ |
| Social network → no use | -0.50 | -8.25 to -0.17 ^**^ | -0.04 | -0.05 to -0.03 ^**^ | -0.06 | -0.08 to -0.05 ^**^ |
| Social network → need | -0.69 | -14.64 to -0.30 ^**^ | -0.04 | -0.06 to -0.03 ^**^ | -0.06 | -0.09 to -0.04 ^**^ |
| Social network → enabling financing | 79.66 | 30.83 to 1360.93 ^**^ | 13.94 | 12.44 to 15.66 ^**^ | 18.45 | 16.36 to 21.13 ^**^ |
| Social network → enabling organisation | 5.33 | 2.09 to 96.01 ^**^ | 0.79 | 0.70 to 0.89 ^**^ | 1.04 | 0.92 to 1.20 ^**^ |
| Social network → registration in primary care | -1.72 | -29.94 to -0.59 ^**^ | -0.30 | -0.34 to -0.26 ^**^ | -0.43 | -0.49 to -0.37 ^**^ |
| Enabling financing → no use | -0.02 | -0.03 to -0.02 ^**^ | 0.00 | -0.01 to 0.00 ^**^ | -0.01 | -0.01 to -0.01 ^**^ |
| Enabling financing → need | 0.00 | -0.01 to 0.00 ^**^ | -0.02 | -0.03 to -0.01 ^**^ | -0.02 | -0.02 to -0.01 ^**^ |
| Enabling organisation → no use | - | - | 0.00 | 0.00 to 0.00 ^**^ | 0.01 | 0.00 to 0.01 ^**^ |
| Enabling organisation → need | - | - | -0.03 | -0.04 to -0.01 ^**^ | -0.04 | -0.05 to -0.03 ^**^ |
| Registration in primary care → no use | -0.03 | -0.04 to -0.02 ^**^ | -0.01 | -0.01 to -0.01 ^**^ | -0.01 | -0.02 to -0.01 ^**^ |
| Registration in primary care → need | 0.01 | 0.00 to 0.02 ^**^ | 0.02 | 0.01 to 0.02 ^**^ | 0.02 | 0.01 to 0.02 ^**^ |
| Education → no use | -0.01 | -0.01 to -0.01 ^**^ | 0.00 | 0.00 to 0.00 ^**^ | -0.01 | -0.01 to -0.01 ^**^ |
| Education → need | -0.01 | -0.01 to -0.01 ^**^ | -0.01 | -0.01 to -0.01 ^**^ | -0.01 | -0.01 to -0.01 ^**^ |
| Education → social network | 0.01 | 0.00 to 0.02 ^**^ | 0.08 | 0.07 to 0.08 ^**^ | 0.06 | 0.05 to 0.07 ^**^ |
| Education → enabling financing | 0.47 | 0.44 to 0.50 ^**^ | 0.84 | 0.82 to 0.85 ^**^ | 0.86 | 0.84 to 0.87 ^**^ |
| Education → enabling organisation | 0.01 | 0.01 to 0.02 ^**^ | 0.04 | 0.04 to 0.04 ^**^ | 0.04 | 0.04 to 0.04 ^**^ |
| Education → registration in primary care | 0.00 | 0.00 to 0.00 ^n.s.^ | -0.02 | -0.02 to -0.02 ^**^ | -0.02 | -0.02 to -0.02 ^**^ |
| Sex → no use | -0.04 | -0.05 to -0.03 ^**^ | -0.01 | -0.02 to -0.01 ^**^ | -0.02 | -0.02 to -0.02 ^**^ |
| Sex → need | 0.05 | 0.04 to 0.06 ^**^ | 0.02 | 0.02 to 0.03 ^**^ | 0.03 | 0.03 to 0.03 ^**^ |
| Sex → social network | -0.13 | -0.27 to -0.01 ^**^ | -0.41 | -0.45 to -0.37 ^**^ | -0.37 | -0.41 to -0.33 ^**^ |
| Sex → enabling financing | -0.04 | -0.23 to 0.17 ^n.s.^ | -1.03 | -1.19 to -0.84 ^**^ | -0.76 | -0.92 to -0.60 ^**^ |
| Sex → enabling organisation | -0.01 | -0.02 to 0.00 ^n.s.^ | -0.01 | -0.01 to 0.00 ^n.s.^ | 0.00 | -0.01 to 0.03 ^n.s.^ |
| Sex → registration in primary care | 0.04 | 0.02 to 0.06 ^**^ | 0.04 | 0.03 to 0.05 ^**^ | 0.04 | 0.03 to 0.04 ^**^ |
| Age → no use | 0.00 | 0.00 to 0.00 ^**^ | 0.00 | 0.00 to 0.00 ^**^ | 0.00 | 0.00 to 0.00 ^**^ |
| Age → social network | 0.00 | -0.01 to 0.00 ^**^ | 0.00 | 0.00 to 0.00 ^**^ | 0.00 | 0.00 to 0.00 ^**^ |
| Age → need | 0.01 | 0.01 to 0.01 ^**^ | 0.01 | 0.01 to 0.01 ^**^ | 0.01 | 0.01 to 0.01 ^**^ |
| Age → enabling financing | 0.06 | 0.06 to 0.07 ^**^ | 0.11 | 0.10 to 0.11 ^**^ | 0.11 | 0.10 to 0.11 ^**^ |
| Age → enabling organisation | 0.00 | 0.00 to 0.00 ^**^ | 0.01 | 0.01 to 0.01 ^**^ | 0.01 | 0.01 to 0.01 ^**^ |
| Age → registration in primary care | 0.00 | 0.00 to 0.00 ^n.s.^ | 0.00 | 0.00 to 0.00 ^**^ | 0.00 | 0.00 to 0.00 ^**^ |

β = bootstrapped non-standardised estimate

^n.s.^ non-significant

^**^ P<0.01
