# Supplementary material for: Utilisation of dental services by Brazilian adults in rural and urban areas: a multi-group structural equation analysis using the Andersen behavioural model
Source: BMC Public Health. 2020 Jun 17;20:953. doi: 10.1186/s12889-020-09100-x (PMC7301519; doi:10.1186/s12889-020-09100-x)
Supplement: Supplementary file 6 — Additional file 6. Direct, indirect and total non-standardised effects on the structural equation model for the interval since the last dental visit outcome in rural and urban contexts. [file 12889_2020_9100_MOESM6_ESM.docx]

Additional file 6. Direct, indirect and total non-standardised effects on the structural equation model for the interval since the last dental visit outcome in rural and urban contexts.

| **Parameter** | **Rural** | | **Urban** | | **Total sample** | |
| --- | --- | --- | --- | --- | --- | --- |
|  | **β** | **Bias-corrected**  **95% CI** | **β** | **Bias-corrected**  **95% CI** | **β** | **Bias-corrected**  **95% CI** |
| **Direct effects** |  |  |  |  |  |  |
| Need → interval since last dental visit | 2.81 | 2.53 to 3.11 ^**^ | 6.61 | 4.69 to 10.90 ^**^ | 6.29 | 4.68 to 9.20 ^**^ |
| Social network → interval since last dental visit | - | - | -6.11 | -11.26 to -3.82 ^**^ | -7.32 | -10.81 to -5.28 ^**^ |
| Social network → need | - | - | 0.28 | 0.15 to 0.47 ^**^ | 0.32 | 0.19 to 0.49 ^**^ |
| Social network → enabling financing | 75.93 | 28.27 to 802.27 ^**^ | 13.57 | 12.09 to 15.12 ^**^ | 17.96 | 15.82 to 20.52 ^**^ |
| Social network → enabling organisation | 3.94 | 1.62 to 101.95 ^**^ | 0.76 | 0.68 to 0.86 ^**^ | 1.00 | 0.88 to 1.15 ^**^ |
| Social network → registration in primary care | -1.75 | -47.90 to -0.60 ^**^ | -0.29 | -0.33 to -0.26 ^**^ | -0.42 | -0.49 to -0.37 ^**^ |
| Enabling financing → interval since last dental visit | -0.07 | -0.08 to -0.06 ^**^ | 0.32 | 0.18 to 0.69 ^**^ | 0.26 | 0.17 to 0.42 ^**^ |
| Enabling financing → need | -0.01 | -0.01 to 0.00 ^**^ | -0.02 | -0.04 to -0.01 ^**^ | -0.02 | -0.03 to -0.01 ^**^ |
| Enabling organisation → interval since last dental visit | - | - | 0.44 | 0.29 to 0.63 ^**^ | 0.61 | 0.46 to 0.83 ^**^ |
| Enabling organisation → need | -0.02 | -0.04 to -0.01 ^**^ | -0.03 | -0.04 to -0.02 ^**^ | -0.04 | -0.05 to -0.03 ^**^ |
| Registration in primary care → interval since last dental visit | -0.19 | -0.24 to -0.13 ^**^ | -0.26 | -0.33 to -0.20 ^**^ | -0.30 | -0.37 to -0.26 ^**^ |
| Registration in primary care → need | 0.01 | 0.00 to 0.02 ^**^ | 0.01 | 0.01 to 0.02 ^**^ | 0.02 | 0.01 to 0.02 ^**^ |
| Education → interval since last dental visit | - | - | 0.17 | 0.10 to 0.31 ^**^ | 0.18 | 0.13 to 0.28 ^**^ |
| Education → need | 0.00 | 0.00 to 0.00 ^**^ | -0.01 | -0.02 to -0.01 ^**^ | -0.01 | -0.02 to -0.01 ^**^ |
| Education → social network | 0.01 | 0.00 to 0.03 ^**^ | 0.08 | 0.07 to 0.08 ^**^ | 0.06 | 0.05 to 0.07 ^**^ |
| Education → enabling financing | -0.53 | -1.37 to -0.20 ^**^ | -0.21 | -0.29 to -0.12 ^**^ | -0.24 | -0.33 to -0.16 ^**^ |
| Education → enabling organisation | -0.04 | -0.07 to -0.02 ^**^ | -0.02 | -0.02 to -0.01 ^**^ | -0.02 | -0.03 to -0.02 ^**^ |
| Education → registration in primary care | 0.02 | 0.01 to 0.04 ^**^ | 0.00 | 0.00 to 0.01 ^*^ | 0.01 | 0.00 to 0.01 ^**^ |
| Sex → interval since last dental visit | -0.31 | -0.36 to -0.26 ^**^ | -2.48 | -4.37 to -1.67 ^**^ | -2.85 | -4.07 to -2.16 ^**^ |
| Sex → need | 0.05 | 0.04 to 0.05 ^**^ | 0.11 | 0.07 to 0.18 ^**^ | 0.13 | 0.09 to 0.18 ^**^ |
| Sex → social network | -0.15 | -0.30 to -0.01 ^**^ | -0.41 | -0.45 to -0.37 ^**^ | -0.37 | -0.42 to -0.33 ^**^ |
| Sex → enabling financing | 11.16 | 7.30 to 19.98 ^**^ | 4.53 | 3.94 to 5.10 ^**^ | 5.90 | 5.27 to 6.54 ^**^ |
| Sex → enabling organisation | 0.58 | 0.39 to 0.99 ^**^ | 0.31 | 0.28 to 0.34 ^**^ | 0.37 | 0.34 to 0.41 ^**^ |
| Sex → registration in primary care | -0.23 | -0.44 to -0.12 ^**^ | -0.08 | -0.10 to -0.07 ^**^ | -0.12 | -0.14 to -0.11 ^**^ |
| Age → interval since last dental visit | - | - | -0.08 | -0.15 to -0.05 ^**^ | -0.08 | -0.13 to -0.06 ^**^ |
| Age → need | 0.01 | 0.01 to 0.01 ^**^ | 0.01 | 0.01 to 0.01 ^**^ | 0.01 | 0.01 to 0.01 ^**^ |
| Age → social network | 0.00 | -0.01 to 0.00 ^**^ | 0.00 | 0.00 to 0.00 ^**^ | 0.00 | 0.00 to 0.00 ^**^ |
| Age → enabling financing | 0.32 | 0.22 to 0.54 ^**^ | 0.13 | 0.12 to 0.15 ^**^ | 0.16 | 0.15 to 0.18 ^**^ |
| Age → enabling organisation | 0.02 | 0.01 to 0.03 ^**^ | 0.01 | 0.01 to 0.01 ^**^ | 0.01 | 0.01 to 0.01 ^**^ |
| Age → registration in primary care | -0.01 | -0.01 to 0.00 ^**^ | 0.00 | 0.00 to 0.00 ^**^ | 0.00 | 0.00 to 0.00 ^**^ |
| **Indirect effects** |  |  |  |  |  |  |
| Social network → interval since last dental visit | -6.34 | -163.96 to -2.46 ^**^ | 4.52 | 2.42 to 9.47 ^**^ | 5.05 | 3.20 to 8.29 ^**^ |
| Social network → need | -0.51 | -10.95 to -0.19 ^**^ | -0.31 | -0.51 to -0.20 ^**^ | -0.37 | -0.52 to -0.26 ^**^ |
| Enabling financing → interval since last dental visit | -0.02 | -0.02 to -0.01 ^**^ | -0.14 | -0.37 to -0.07 ^**^ | -0.11 | -0.21 to -0.06 ^**^ |
| Enabling organisation → interval since last dental visit | -0.06 | -0.10 to -0.02 ^**^ | -0.20 | -0.37 to -0.10 ^**^ | -0.24 | -0.42 to -0.13 ^**^ |
| Registration in primary care → interval since last dental visit | 0.03 | 0.01 to 0.05 ^**^ | 0.10 | 0.06 to 0.17 ^**^ | 0.10 | 0.07 to 0.16 ^**^ |
| Education → interval since last dental visit | -0.05 | -0.05 to -0.05 ^**^ | -0.24 | -0.38 to -0.17 ^**^ | -0.26 | -0.35 to -0.20 ^**^ |
| Education → need | 0.00 | 0.00 to 0.00 ^**^ | 0.00 | 0.00 to 0.01 ^n.s.^ | 0.00 | 0.00 to 0.01 ^n.s.^ |
| Education → enabling financing | 1.00 | 0.67 to 1.87 ^**^ | 1.04 | 0.96 to 1.13 ^**^ | 1.09 | 1.01 to 1.18 ^**^ |
| Education → enabling organisation | 0.05 | 0.03 to 0.09 ^**^ | 0.06 | 0.05 to 0.06 ^**^ | 0.06 | 0.06 to 0.07 ^**^ |
| Education → registration in primary care | -0.02 | -0.04 to -0.01 ^**^ | -0.02 | -0.03 to -0.02 ^**^ | -0.03 | -0.03 to -0.02 ^**^ |
| Sex → interval since last dental visit | 0.13 | 0.11 to 0.16 ^**^ | 2.31 | 1.50 to 4.20 ^**^ | 2.68 | 1.99 to 3.89 ^**^ |
| Sex → need | 0.00 | 0.00 to 0.00 ^n.s.^ | -0.09 | -0.16 to -0.05 ^**^ | -0.10 | -0.16 to -0.06 ^**^ |
| Sex → enabling financing | -11.28 | -19.95 to -7.43 ^**^ | -5.59 | -6.18 to -5.06 ^**^ | -6.73 | -7.41 to -6.13 ^**^ |
| Sex → enabling organisation | -0.59 | -1.00 to -0.40 ^**^ | -0.31 | -0.35 to -0.28 ^**^ | -0.38 | -0.42 to -0.34 ^**^ |
| Sex → registration in primary care | 0.26 | 0.15 to 0.48 ^**^ | 0.12 | 0.11 to 0.14 ^**^ | 0.16 | 0.14 to 0.18 ^**^ |
| Age → interval since last dental visit | 0.02 | 0.02 to 0.02 ^**^ | 0.09 | 0.06 to 0.16 ^**^ | 0.10 | 0.07 to 0.14 ^**^ |
| Age → need | 0.00 | 0.00 to 0.00 ^**^ | 0.00 | 0.00 to 0.00 ^**^ | 0.00 | 0.00 to 0.00 ^**^ |
| Age → enabling financing | -0.25 | -0.49 to -0.15 ^**^ | -0.02 | -0.04 to -0.01 ^**^ | -0.06 | -0.08 to -0.04 ^**^ |
| Age → enabling organisation | -0.01 | -0.02 to -0.01 ^**^ | 0.00 | 0.00 to 0.00 ^**^ | 0.00 | 0.00 to 0.00 ^**^ |
| Age → registration in primary care | 0.01 | 0.00 to 0.01 ^**^ | 0.00 | 0.00 to 0.00 ^**^ | 0.00 | 0.00 to 0.00 ^**^ |
| **Total effects** |  |  |  |  |  |  |
| Need → interval since last dental visit | 2.81 | 2.53 to 3.11 ^**^ | 6.61 | 4.69 to 10.90 ^**^ | 6.29 | 4.60 to 9.09 ^**^ |
| Social network → interval since last dental visit | -6.34 | -163.96 to -2.46 ^**^ | -1.59 | -1.85 to -1.34 ^**^ | -2.27 | -2.80 to -1.93 ^**^ |
| Social network → need | -0.51 | -10.95 to -0.19 ^**^ | -0.04 | -0.05 to -0.02 ^**^ | -0.05 | -0.07 to -0.03 ^**^ |
| Social network → enabling financing | 75.93 | 28.27 to 802.27 ^**^ | 13.57 | 12.09 to 15.12 ^**^ | 17.96 | 15.91 to 20.60 ^**^ |
| Social network → enabling organisation | 3.94 | 1.62 to 101.95 ^**^ | 0.76 | 0.68 to 0.86 ^**^ | 1.00 | 0.89 to 1.17 ^**^ |
| Social network → registration in primary care | -1.75 | -47.90 to -0.60 ^**^ | -0.29 | -0.33 to -0.26 ^**^ | -0.42 | -0.49 to -0.37 ^**^ |
| Enabling financing → interval since last dental visit | -0.08 | -0.10 to -0.08 ^**^ | 0.18 | 0.11 to 0.35 ^**^ | 0.14 | 0.09 to 0.22 ^**^ |
| Enabling financing → need | -0.01 | -0.01 to 0.00 ^**^ | -0.02 | -0.04 to -0.01 ^**^ | -0.02 | -0.02 to -0.01 ^**^ |
| Enabling organisation → interval since last dental visit | -0.06 | -0.10 to -0.02 ^**^ | 0.25 | 0.15 to 0.35 ^**^ | 0.37 | 0.26 to 0.51 ^**^ |
| Enabling organisation → need | -0.02 | -0.04 to -0.01 ^**^ | -0.03 | -0.04 to -0.02 ^**^ | -0.04 | -0.05 to -0.03 ^**^ |
| Registration in primary care → interval since last dental visit | -0.16 | -0.21 to -0.11 ^**^ | -0.16 | -0.19 to -0.14 ^**^ | -0.19 | -0.22 to -0.17 ^**^ |
| Registration in primary care → need | 0.01 | 0.00 to 0.02 ^**^ | 0.01 | 0.01 to 0.02 ^**^ | 0.02 | 0.01 to 0.02 ^**^ |
| Education → interval since last dental visit | -0.05 | -0.05 to -0.05 ^**^ | -0.07 | -0.08 to -0.07 ^**^ | -0.08 | -0.08 to -0.07 ^**^ |
| Education → need | -0.01 | -0.01 to -0.01 ^**^ | -0.01 | -0.01 to -0.01 ^**^ | -0.01 | -0.01 to -0.01 ^**^ |
| Education → social network | 0.01 | 0.00 to 0.03 ^**^ | 0.08 | 0.07 to 0.08 ^**^ | 0.06 | 0.05 to 0.07 ^**^ |
| Education → enabling financing | 0.47 | 0.44 to 0.50 ^**^ | 0.84 | 0.82 to 0.85 ^**^ | 0.85 | 0.84 to 0.87 ^**^ |
| Education → enabling organisation | 0.01 | 0.01 to 0.02 ^**^ | 0.04 | 0.04 to 0.04 ^**^ | 0.04 | 0.04 to 0.04 ^**^ |
| Education → registration in primary care | 0.00 | 0.00 to 0.00 ^**^ | -0.02 | -0.02 to -0.02 ^**^ | -0.02 | -0.02 to -0.02 ^**^ |
| Sex → interval since last dental visit | -0.18 | -0.23 to -0.13 ^**^ | -0.17 | -0.19 to -0.15 ^**^ | -0.18 | -0.20 to -0.16 ^**^ |
| Sex → need | 0.05 | 0.04 to 0.05 ^**^ | 0.02 | 0.02 to 0.03 ^**^ | 0.03 | 0.02 to 0.03 ^**^ |
| Sex → social network | -0.15 | -0.30 to -0.01 ^**^ | -0.41 | -0.45 to -0.37 ^**^ | -0.37 | -0.41 to -0.33 ^**^ |
| Sex → enabling financing | -0.13 | -0.31 to 0.09 ^n.s.^ | -1.06 | -1.23 to -0.89 ^**^ | -0.84 | -0.98 to -0.67 ^**^ |
| Sex → enabling organisation | -0.01 | -0.02 to 0.00 ^n.s.^ | -0.01 | -0.01 to 0.00 ^n.s.^ | -0.01 | -0.01 to 0.00 ^n.s.^ |
| Sex → registration in primary care | 0.03 | 0.02 to 0.05 ^**^ | 0.04 | 0.03 to 0.05 ^**^ | 0.04 | 0.03 to 0.04 ^**^ |
| Age → interval since last dental visit | 0.02 | 0.02 to 0.02 ^**^ | 0.01 | 0.01 to 0.01 ^**^ | 0.01 | 0.01 to 0.01 ^**^ |
| Age → social network | 0.00 | -0.01 to 0.00 ^**^ | 0.00 | 0.00 to 0.00 ^**^ | 0.00 | 0.00 to 0.00 ^**^ |
| Age → need | 0.01 | 0.01 to 0.01 ^**^ | 0.01 | 0.01 to 0.01 ^**^ | 0.01 | 0.01 to 0.01 ^**^ |
| Age → enabling financing | 0.07 | 0.06 to 0.07 ^**^ | 0.11 | 0.10 to 0.11 ^**^ | 0.11 | 0.10 to 0.11 ^**^ |
| Age → enabling organisation | 0.00 | 0.00 to 0.00 ^**^ | 0.01 | 0.01 to 0.01 ^**^ | 0.01 | 0.01 to 0.01 ^**^ |
| Age → registration in primary care | 0.00 | 0.00 to 0.00 ^n.s.^ | 0.00 | 0.00 to 0.00 ^**^ | 0.00 | 0.00 to 0.00 ^**^ |

β = bootstrapped non-standardised estimate

^n.s.^ non-significant

^*^ P<0.05

^**^ P<0.01
